# Plasticity of ventricle position after heart looping in heterotaxy with right isomerism

**DOI:** 10.1126/sciadv.ads8192

**Published:** 2025-09-19

**Authors:** Audrey Desgrange, Emeline Perthame, Carmen Marchiol, Daphné Madec, Laurent Guillemot, Marie-Amandine Chabry, Mohamed El Beheiry, Jean-Baptiste Masson, Olivier Raisky, Ségolène Bernheim, Lucile Houyel, Sigolène M. Meilhac

**Affiliations:** ^1^ Université Paris Cité, Paris 75006, France.; ^2^ Imagine – Institut Pasteur, Unit of Heart Morphogenesis, INSERM UMR1163, Paris 75015, France.; ^3^ Institut Pasteur, Bioinformatics and Biostatistics Hub, Paris 75015, France.; ^4^ INSERM U1016 and CNRS UMR8104, Institut Cochin, Paris 75014, France.; ^5^ Unité Médico-Chirurgicale de Cardiologie Congénitale et Pédiatrique, M3C-Necker, Hôpital Universitaire Necker-Enfant-Malades, APHP, Paris 75015, France.; ^6^ Sorbonne Université, Institut Curie, PSL Research University, CNRS UMR 168 Physico-Chimie Curie, Paris 75005, France.; ^7^ Institut Pasteur, CNRS UMR 3571, Decision and Bayesian Computation, Paris 75015, France.; ^8^ Epiméthée, INRIA, Paris, France.

## Abstract

The heart functions in two parallel but asymmetric circulations, driven by the right and left ventricles. In the heterotaxy syndrome, abnormal left-right patterning leads to a spectrum of severe congenital heart defects, including ventricle malposition. A postulate anchored in the clinical nomenclature assumes that the looping direction of the embryonic heart tube determines ventricle position at birth. However, this has not been demonstrated experimentally. Here, we performed a unique longitudinal analysis of heterotaxy with right isomerism, using multimodality imaging of *Nodal* mouse mutants. Using direct correlations and advanced statistics, we dissected the contribution of heart looping variations to specific structural heart malformations, and uncovered unexpected plasticity of ventricle position after looping in 30% of revertant samples. Genetic tracing and topological associations show that plasticity involves a further step of heart remodeling at E13.5, rather than molecular reprogramming. Human patient scans are consistent with ventricle plasticity and suggest association with poorer prognosis. Our work reveals distinct asymmetric events shaping organs.

## Teaser

The left/right position of ventricles needs to be maintained after heart looping, an unexpected step affected in heterotaxy.

## INTRODUCTION

Visceral organs have an asymmetric shape and/or position. The left-right partition of the heart is an essential aspect of its function, to separate oxygenated from carbonated blood. Cardiac ventricles are analogous segments, yet with anatomical differences underlying their distinct functions of driving the systemic or pulmonary circulations. Ventricles develop as distinct entities already in the early embryonic heart tube, but are immature at this stage, with incorrect position and connection to other cardiac segments (*1*). In the heterotaxy syndrome, in which left-right patterning is impaired, heart dysfunction is the major prognostic factor in patients (*2*).

Heterotaxy syndrome is a rare laterality defect, affecting 8 of 100,000 births and encompassing a broad spectrum of defects in visceral organs (*3*, *4*). The left-right asymmetric position or shape of organs can be defective, and laterality can be discordant between organs or organ segments. In most cases (90%), heterotaxy is associated with congenital heart defects, mainly complex, which obstruct blood flow or compromise oxygen supply when the left and right cardiac circuits are not completely separated (*5*). The origin of heterotaxy in patients is associated with variations in genes involved in the formation of the node, in ciliary genes or in genes of the NODAL pathway (*2*, *6*). Studies in the mouse model over the last three decades have demonstrated the underlying mechanism, by showing how the symmetry of the early embryo is broken in the node (also referred to as the left-right organizer): A leftward fluid flow generated by motile cilia and sensed by immotile cilia induces left-sided NODAL signaling in the lateral plate mesoderm, which controls the asymmetric morphogenesis of visceral organs (*7*–*11*). However, the broad phenotypic spectrum of heterotaxy cannot be explained by a single asymmetric event and thus its origin has remained enigmatic. We hypothesize that the spectrum of defects in heterotaxy reflects embryological mechanisms.

Brown and Wolpert (*12*) had proposed that asymmetric organogenesis requires an additional step after symmetry breaking, during which an organ-specific random generator of asymmetry shapes organs specifically, while being coordinated by the overall left-right bias of the organizer. In the context of the heart, we have shown how this concept manifests as a buckling mechanism during the rightward looping of the embryonic heart tube. The tubular primordium of the heart is initially straight at embryonic day (E) 8.5e. We have demonstrated that the heart tube elongates, while the distance between the arterial and venous poles remains fixed. This induces a buckling mechanism, able to deform the heart tube (*13*). We have further shown that NODAL is not required for buckling, but rather to bias it, thus controlling the shape of the embryonic heart loop at E9.5 (*14*). In keeping with the helical shape of the looped heart tube in the mouse, we identified two asymmetries in the arterial and venous poles of the heart, able to deform the tube in 3D (*13*, *14*). Upon mesoderm inactivation of *Nodal*, these two asymmetries are randomly oriented, resulting in four classes of abnormal heart loop shapes (*14*). How specific configurations of the heart loop determine the heart structure at birth is an open question that we address here.

Mouse models of heterotaxy, which exhibit structural heart defects at birth, also show abnormal heart looping in the embryo, indicating the importance of heart looping for heart morphogenesis (*9*, *14*–*18*). *Nodal* is transiently expressed in heart precursors, before the formation of the heart tube (*14*). NODAL signaling has been considered so far to regulate heart morphogenesis by controlling heart looping, which is the first asymmetric process during heart development. When anatomists observed inverted ventricle positions in human heart specimens (*19*, *20*), they proposed that it could result from a reversed direction of embryonic heart looping. Van Praagh (*21*, *22*) based his segmental analysis, widely used by clinicians, on this mechanistic hypothesis and described the laterality of ventricles in the mature heart, as D-LOOP (*dexter*, right), generally when the anatomic right ventricle is normally on the right side of the anatomic left ventricle, and L-LOOP (*laevus*, left) when it lies on the left side. The clinical nomenclature thus postulates that ventricle position is set during heart looping. However, this has never been demonstrated experimentally. This is challenging and requires both a longitudinal analysis, to overcome interindividual phenotypic variations, as well as expertise in the diagnosis of congenital heart defects.

Van Praagh (*23*) has established a segmental approach to diagnose congenital heart defects, which uncouples the identity of cardiac segments (e.g., morphological right ventricle), from their position (e.g., on the left side) and their connections to the other segments (i.e., atria and great arteries). To diagnose heterotaxy, several cardiac features are thus recorded independently, including also potential deviations in the size balance of analogous segments. This assessment is completed by the position of the heart in the thoracic cavity, and description of laterality defects in other visceral organs (*3*, *23*, *24*). To investigate the origin of heterotaxy defects, we have previously established a multimodality imaging pipeline in the mouse, able to track single individuals and correlate their phenotype at two stages of development (*24*).

Here, in an interdisciplinary approach combining multimodality imaging of developing mouse mutants and clinical-like diagnosis, we perform an extensive longitudinal analysis of heterotaxy. We use *Nodal* conditional mutants as a model of heterotaxy with right isomerism. On the basis of advanced statistical analyses, we uncover how the embryonic heart loop shape can be predictive of features of the final heart structure. However, we find that heart looping is a partial predictor of heart structure. In particular, the hypothesis implied by the clinical nomenclature of “D-LOOP” and “L-LOOP” to describe the laterality of ventricles is not validated. We detect unexpected plasticity of ventricle position after heart looping in heterotaxy. Revertants, which had a leftward embryonic heart looping, but display a D-LOOP configuration at birth, are enriched in ventricular defects. Genetic tracing, molecular analyses, and topological association support a mechanism of heart remodeling after the looping stage, rather than molecular reprogramming of ventricles. Overall, our study reveals an extra step of asymmetry at E13.5. Consistent observations in patients indicate that ventricle plasticity is a potential risk factor to consider. This contributes to a better understanding of the broad phenotypic spectrum of heterotaxy, which has been overlooked by the consideration of a single asymmetric event in the left-right organizer.

## RESULTS

### Longitudinal analysis of heart defects in heterotaxy with right isomerism

**Fig. 1.**
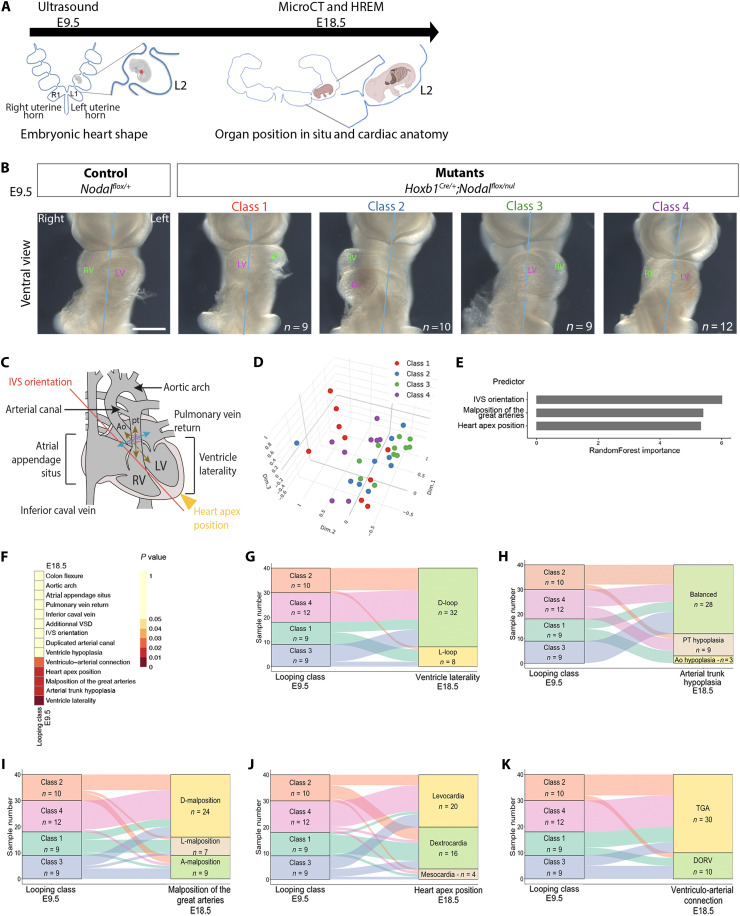
Individual association between the embryonic heart loop shape and the mature heart structure in *Nodal* mutants. (**A**) Multimodality imaging pipeline used for longitudinal analysis of individuals. In utero ultrasound imaging at E9.5 assessed the shape of the embryonic heart loop. Micro-CT and HREM of E18.5 fetuses identified laterality defects of visceral organs and congenital heart defects, respectively. Individuals were tracked by uterine horn position (e.g., L2 = second embryo in the left horn). (**B**) Bright-field images of *Nodal* control and mutant embryos in a control cohort dissected at E9.5, showing their embryonic heart looping class with randomized distribution (see fig. S1A and table S8). (**C**) Schematic representation of anatomical features in E18.5 hearts, including arterial valves (pink), the plane used to determine great artery malposition (blue double arrowheads), ventriculo-arterial connections (brown double arrowheads). (**D**) Scatter plot of samples from a multiple correspondence analysis of 11 parameters in E18.5 *Nodal* mutant hearts (*n* = 37, see table S4), color coded for the associated looping class at E9.5. (**E**) Supervised classification of the looping class at E9.5, with a random forest model on 14 parameters at E18.5 (*n* = 40 mutants, see table S4), identifies three main phenotypic signatures. (**F**) Pairwise associations between the looping class at E9.5 and the indicated E18.5 features, ordered by *P* values. (**G** to **K**) Sankey plots showing the direct association between the looping class at E9.5 and specific E18.5 features: ventricle laterality (G), arterial trunk hypoplasia (H), malposition of the great arteries (I), heart apex position (J), and ventriculo-arterial connection (K). Ao, aorta; DORV, double-outlet right ventricle; IVS, interventricular septum; L, left; LV, left ventricle; PT, pulmonary trunk; R, right; RV, right ventricle; TGA, transposition of the great arteries; VSD, ventricular septal defect. Scale bar, 200 μm. See also figs. S1 to S3 and movies S1 to S3.

To investigate the mechanisms of heterotaxy, we used a mouse model that we have previously characterized, in which the left determinant NODAL is inactivated throughout the lateral plate mesoderm (*14*). *Nodal^flox/null^*;*Hoxb1^Cre/+^* conditional mutants at E18.5 display heterotaxy with right isomerism of the lungs and bronchi, full penetrance of atrioventricular septal defects, abnormal spatial position (malposition) of the great arteries, and abnormal ventriculo-arterial connections. However, they have variable defects in colon flexure, heart apex position, ventricle position and size, atrial situs, arterial trunk size, and venous return insertion (table S1). At E9.5, *Nodal* mutants fall into four classes of looping defects, with equal frequency but distinct shapes (Fig. 1B and figs. S1A and S2, D to F). To test the hypothesis that the heterogeneity of congenital heart defects results from looping variations, we performed a longitudinal analysis, correlating the embryonic and perinatal phenotypes. We used a multimodality imaging pipeline (Fig. 1A), combining ultrasound imaging of embryos in utero (movie S1) with in-depth heterotaxy phenotyping of the fetus by micro–computed tomography (CT) of visceral organs in situ (movie S2) and by high-resolution episcopic microscopy (HREM) of cardiac anatomy (movie S3). We have thus generated a unique cohort of 43 *Nodal^flox/null^*;*Hoxb1^Cre/+^* mutants and 22 littermate controls (table S2), in which each individual has been tracked on the basis of its position in the uterine horns (table S3) and phenotyped both at E9.5 and E18.5 (Fig. 1, A to C, and figs. S1 and S3). We controlled accurate diagnosis by ultrasound imaging at E9.5 in a cohort dissected immediately after ultrasound imaging (fig. S2). We did not observe any specific in utero death of mutants, which were collected with a Mendelian ratio (fig. S1J). Twenty-one parameters were overall annotated (Fig. 1C; fig. S1, B to I; and table S4), which are all qualitative, but three based on quantitative measures: orientation of the interventricular septum, ventricle hypoplasia, and arterial trunk hypoplasia (fig. S3). As a user-friendly community resource, we have created a web interface to interrogate these data, accessible at https://htx.pasteur.cloud.

### The embryonic heart loop shape is a partial predictor of congenital heart defects associated with heterotaxy

We showed previously that all classes of embryonic heart loop in *Nodal* mutants were abnormal, including a lower degree of arterial pole rotation and of leftward displacement of the venous pole (*14*), which may reflect the full penetrance at E18.5 of malposition and misconnections of the great arteries and of atrioventricular septal defects. To understand whether variations in the shape of the embryonic heart loop between the four classes predispose to a specific congenital heart defect, we first performed a multiple correspondence analysis (MCA) on the 11 most variable cardiac phenotypic features at E18.5. This does not graphically highlight any obvious clustering, and does not segregate the associated heart looping classes (Fig. 1D). We then conducted a supervised classification with a random forest model on 14 variables to quantify their prediction capabilities. The best model was able to correctly predict the looping class of 48% of samples, which is significantly better (*P* = 0 for a permutation test with 5000 permutations) compared to a random classification (23% in average). The variables that were most important to determine the looping class are the orientation of the interventricular septum, the spatial position of the great arteries and the position of the heart apex (Fig. 1E). We analyzed this in more detail by testing pairwise associations between the looping classes and the variable phenotypic features at E18.5 (Fig. 1F): This highlights additional associated variables. The association with ventricle laterality was highly significant (*P* = 0.008): Looping class 4 is only associated with normal D-LOOP, as well as most of class 2 (Fig. 1G). The association with arterial trunk hypoplasia was significant (*P* = 0.01): Looping class 3 is always associated with a balanced diameter of the arterial trunks, whereas hypoplastic aorta is only associated with looping class 1 (Fig. 1H). The association with the position of the great arteries was significant (*P* = 0.01): Looping class 4 is mainly associated with a D-malposition and never with L-malposition (Fig. 1I). The association with the position of the heart apex was significant (*P* = 0.018): Looping class 1 is mainly associated with dextrocardia, whereas looping class 4 is mainly associated with normal levocardia (Fig. 1J). The association with ventriculo-arterial connections was significant (*P* = 0.03): Looping class 4 is only associated with transposition of the great arteries (Fig. 1K). Overall, looping classes 1 and 4 are the strongest determinants of cardiac structure at E18.5. Looping class 4, which is the closest to the control shape, tends to have milder congenital heart defects and is enriched in the {S;D;D} segmental nomenclature of Van Praagh (*23*). We conclude that the spectrum of congenital heart defects in heterotaxy with right isomerism is only partially explained by the shape of the embryonic heart loop, which indicates that left-right asymmetry of the heart is not fully established at E9.5 and that there are additional morphogenetic steps apart from heart looping that depend on NODAL signaling.

### Heart looping direction is not the only determinant of ventricle position in heterotaxy

**Fig. 2.**
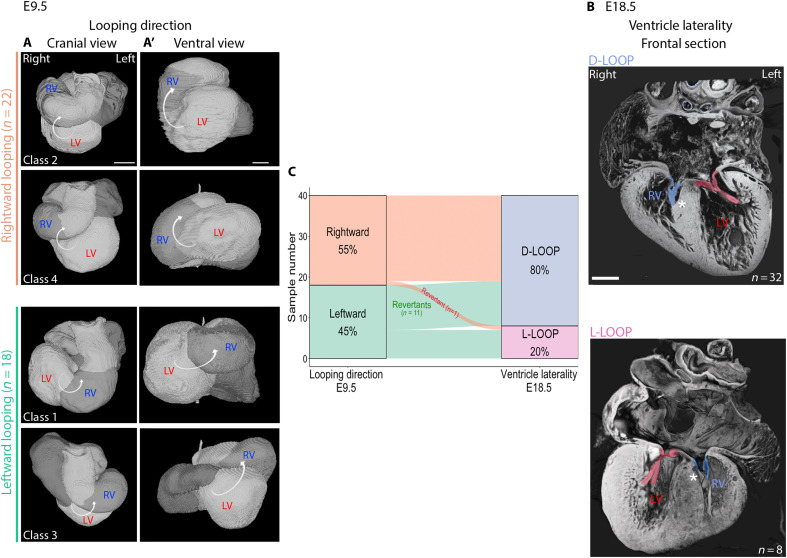
Plasticity of ventricle laterality after heart looping in *Nodal* mutants. (**A** and **A’**) 3D reconstructions of the segmented heart in *Nodal* mutants at E9.5, seen in a cranial (A) and ventral (A’) view and aligned according to the notochord. The white arrow indicates the looping direction. See also fig. S2. (**B**) Frontal section from HREM 3D reconstructions of E18.5 hearts in *Nodal* mutants, highlighting the distinct anatomy of the atrioventricular valves, diagnostic of the anatomic right and left ventricles. In the D-LOOP configuration shown here, the anatomic right ventricle lies on the right side of the left ventricle, whereas it is on its left side in L-LOOP. The tricuspid valve is colored in blue and the mitral valve in red. The asterisk indicates the septal attachment of the tricuspid valve. (**C**) Sankey plot showing the direct association between the direction of embryonic heart looping at E9.5 and later ventricle laterality in the same heart tracked at E18.5. LV, left ventricle; RV, right ventricle. Scale bars, 100 μm (A) and 400 μm (B).

The laterality of ventricles is thought to be determined by the direction of embryonic heart looping, as reflected by the clinical nomenclature of D-LOOP and L-LOOP when the anatomic right ventricle is positioned on the right and left side of the anatomic left ventricle, respectively, or in case of ventricle malposition, based on the principle of handedness of the tripartite ventricle (*25*). However, the underlying hypothesis has never been demonstrated experimentally. We investigated this correlation in our cohort of *Nodal* mutants (Fig. 2). At E9.5, 55% mutant hearts have a rightward looping, corresponding to classes 2 and 4, and 45% mutant hearts have a leftward looping, corresponding to classes 1 and 3. However at E18.5, 80% of mutant hearts have a D-LOOP and 20% a L-LOOP, showing a deficit of L-LOOP compared to expectations. When we performed pairwise associations, we found that 70% of samples follow the logics of rightward looping giving rise to D-LOOP and vice versa. However, unexpectedly, 30% of samples (12 of 40) were identified as revertants (Fig. 2C). This demonstrates that the position of ventricles in heterotaxy is not fully determined by the direction of heart looping, and that ventricle position can be plastic after heart looping.

### Consistency of genetic tracing, molecular and anatomic identities of ventricles rules out reprogramming

**Fig. 3.**
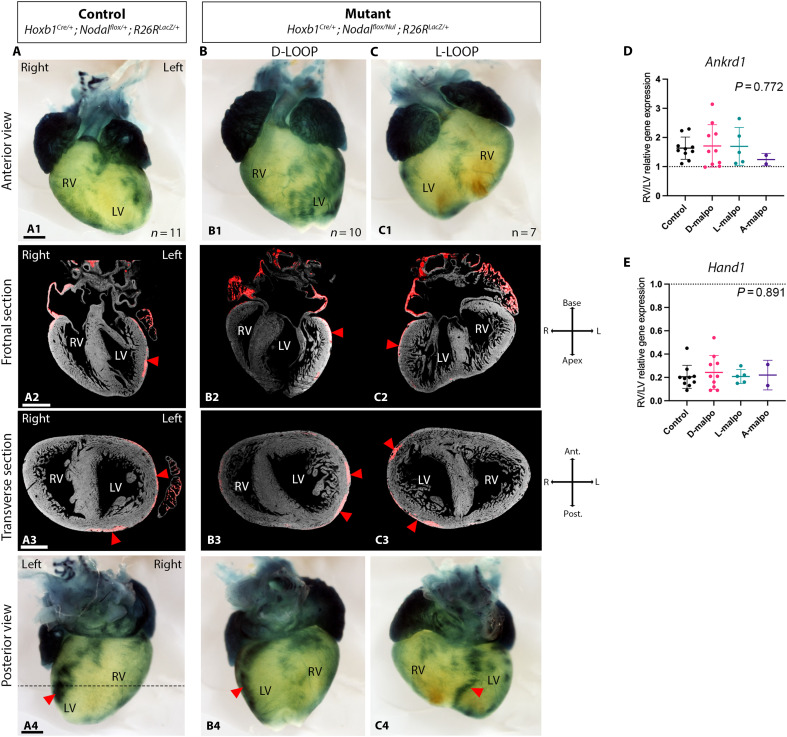
Genetic tracing and molecular identities of ventricles in *Nodal* mutants. (**A** to **C**) *Hoxb1^Cre^* genetic tracing in control (A) and *Nodal* mutant hearts at E18.5 with D-LOOP (B) or L-LOOP (C). (a1-c1 and a4-c4) Bright-field images seen in anterior (a1-c1) and posterior (a4-c4) views. (a2-c2 and a3-c3) HREM 3D reconstructions, showing the histology (gray) and the β-galactosidase staining (red), in frontal (a2-c2) and transverse (a3-c3) sections. The dotted line in a4 indicates the plane of the transverse section in a3-c3. In a control heart (A) *Hoxb1^Cre^* genetic tracing labels the dorsal left ventricle, in addition to the atria and arterial trunks. In mutant hearts, the right (RV) and left (LV) ventricles are annotated on the basis of their anatomy (a2-c2). The main ventricular β-galactosidase–positive cluster is indicated by red arrowheads. (**D** and **E**) Relative expression of the right ventricular marker *Ankrd1* (D) and the left ventricular marker *Hand1* (E) between paired ventricles in controls and mutants with different configurations. Expression is measured by RT-qPCR on anatomically determined ventricles. Expression is not significantly different between samples (ANOVA, *n* = 10 controls, 10 mutants with D-malposition, 5 with L-malposition, 2 with A-malposition of the great arteries). Ant., anterior; L, left; LV, left ventricle; Post., posterior; R, right; RV, right ventricle. Scale bars: 500 μm (a1-c2 and a4-c4) and 400 μm (a3-c3). See also fig. S4 and table S8.

We hypothesized that ventricle position could change either because of a molecular reprogramming of ventricles or due to a further morphological remodeling of the heart. We took advantage of the presence of the Cre recombinase to perform genetic tracing in *Nodal* conditional mutants. *Hoxb1* is expressed in the mesoderm at the onset of gastrulation (*26*), but no longer in the developing heart (fig. S4A). It is not affected by the loss of *Nodal* (fig. S4B). Genetic tracing of heart precursors expressing *Hoxb1* had previously shown some labeling of the left, but not right ventricle (*27*) (see also fig. S4C). Thus, we performed genetic tracing of the left ventricle in a distinct cohort of *Nodal^flox/null^*;*Hoxb1^Cre/+^;R26R^lacZ/+^* mutants, compared to littermate controls at E18.5. In all controls, β-galactosidase staining was mainly detected in the dorsolateral left ventricle (Fig. 3A). In *Nodal^flox/null^*;*Hoxb1^Cre/+^;R26R^lacZ/+^* mutants, the staining was similarly mainly detected in the anatomic left ventricle in D-LOOP samples (Fig. 3B). In mutant samples with L-LOOP, the main staining was still associated with the anatomic left ventricle, but it was more dorsal, close to the interventricular septum (Fig. 3C). These observations show that the anatomic identity of the left ventricle is consistent with its embryonic origin. In addition, *Nodal* is not expressed in the heart after E8.5e, and genetic tracing with the *NodalASE-lacZ* transgene, shows barely any contribution of *Nodal* positive cardiac precursors to the ventricles (fig. S4, D to F). We next assessed the molecular signature of ventricles at E18.5. We identified differential enrichment of *Ankrd1* and *Hand1* expression, in the right and left ventricle, respectively. We did not detect any change in these specific markers, in mutant hearts with different anatomical configurations (Fig. 3, D and E). Gene expression aligned with ventricle anatomy. Together, our observations indicate that *Nodal* does not directly reprogram ventricle identity, but rather modulates heart morphogenesis by acting outside ventricles. Our observations thus rule out a mechanism of ventricle reprogramming in revertants.

### Topological association between the laterality of ventricles and of great arteries indicates an overall heart remodeling after embryonic looping

**Fig. 4.**
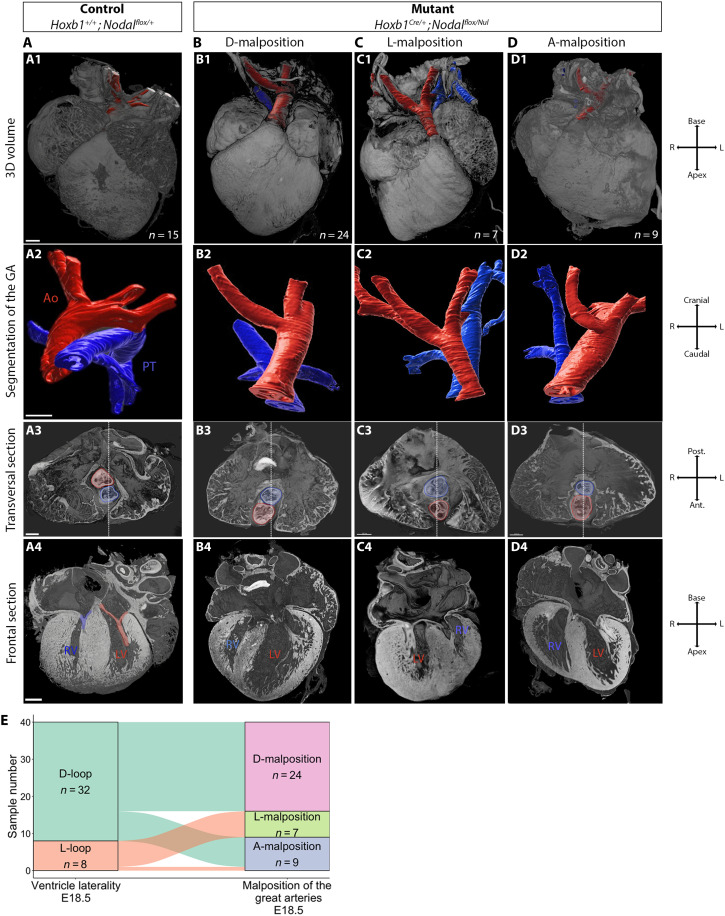
Individual association between the laterality of the great arteries and that of ventricles in *Nodal* mutants. (**A** to **D**) HREM 3D images of hearts at E18.5 in control (A) and *Nodal* mutants [(B) to (D)]. (a1-d1) 3D rendering seen in an anterior view, with the ascending aorta segmented in red and the pulmonary trunk in blue. (a2-d2) Focus on the segmented great arteries (GA) of the same hearts, showing normal spiraling of the ascending aorta over the pulmonary trunk in controls and parallel great arteries in mutants. (a3-d3) Transverse sections of the same hearts at the level of arterial valves showing the relative position of the ascending aorta and pulmonary trunk. The white dotted line indicates the midline of the pulmonary trunk. The ascending aorta is right-sided (b3, D-malposition), left-sided (c3, L-malposition), or centered as the pulmonary trunk (d3, A-malposition). (a4-d4) Frontal sections of the same hearts showing ventricle laterality. The tricuspid valve is colored in blue and mitral valve in red (a4). (**E**) Sankey plot showing the direct association between ventricle laterality and malposition of the great arteries in the same heart. Ant., anterior; Ao, aorta; L, left; LV, left ventricle; PT, pulmonary trunk; Post., posterior; R, right; RV, right ventricle. Scale bars, 300 μm.

The laterality of ventricles has been reported in human specimens to correlate with that of the great arteries (*22*). We investigated this in the mouse cohort. All *Nodal* mutant hearts have malposition of the great arteries, with the ascending aorta being anterior to the pulmonary trunk. Depending on the laterality of the ascending aorta compared to the pulmonary trunk, the malposition is diagnosed as D (right-sided ascending aorta) or L (left-sided ascending aorta) or A when the laterality is undetermined so that the aorta is strictly anterior (Fig. 4, A to D). Pairwise associations with the laterality of ventricles are highly significant (*P* = 2 × 10^−8^): Samples with D-LOOP never have a L-malposition of the great arteries and in reverse, samples with L-LOOP never have a D-malposition of the great arteries (Fig. 4E), including in revertants (table S4). Thus, when ventricles change their position in revertants, this remains consistent with the laterality of the great arteries. This shows topological constraints and supports a mechanism of overall remodeling of the heart. We conclude that ventricle plasticity in heterotaxy is associated with remodeling of the heart after embryonic heart looping rather than molecular reprogramming.

### The kinetics of ventricle position distribution shows remodeling at E13.5

**Fig. 5.**
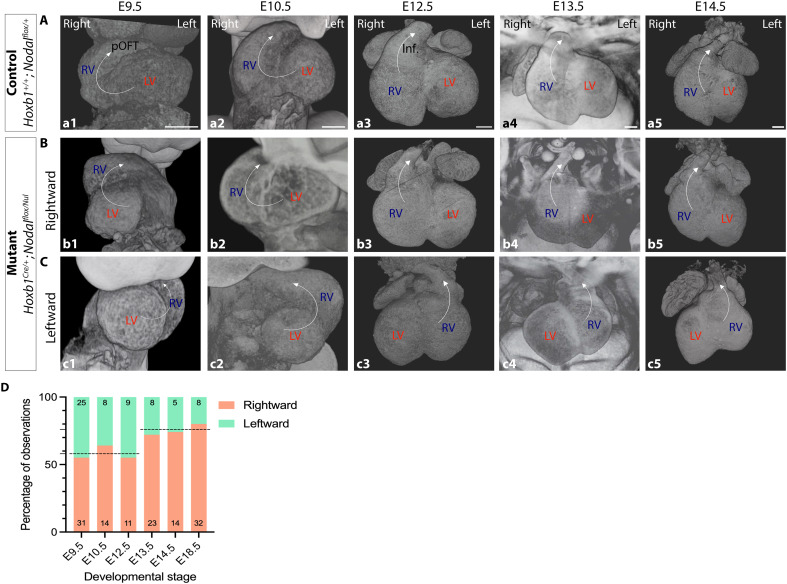
Staging of the remodeling leading to ventricle position reversion. (**A** to **C**) Ventral (E9.5 to E10.5) and anterior views of 3D HREM images of the heart in control (a1-a5) and *Nodal* mutants (b1-c5), at the indicated sequential stages. The right ventricle is defined on the basis of its continuity with the proximal outflow tract (pOFT, E9.5 to E10.5) and its derivative, the infundibulum (Inf., E12.5 to E14.5). Examples of rightward (B) and leftward (C) mutant configurations are shown, highlighted by white arrows. (**D**) Frequency of rightward and leftward configurations at the indicated developmental stages, reflecting heart looping at E9.5 and ventricle laterality at E18.5. LV, left ventricle; RV, right ventricle. Scale bars, 200 μm

To get insight into the time window when heart remodeling occurs, we collected *Nodal* mutants at sequential stages, between E9.5 and E18.5, and quantified the frequency of rightward and leftward configurations (Fig. 5, A to C). Between E9.5 and E12.5, we detected a constant proportion of 58% ± 5 of rightward. However, from E13.5 to E18.5, the distribution abruptly shifted to 76% ± 4 rightward (Fig. 5D). This demonstrates that heart remodeling occurs between E12.5 and E13.5.

### Reversal of ventricle laterality is associated with increased ventricular defects

**Fig. 6.**
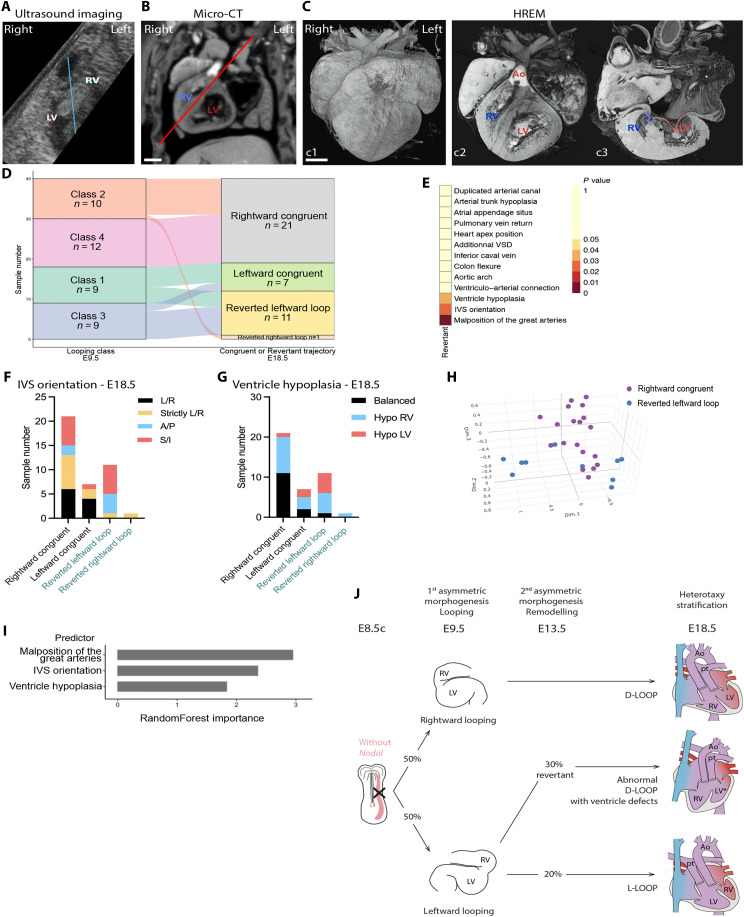
Characteristics of revertants, undergoing ventricle position plasticity. (**A**) In utero imaging of a revertant *Nodal* mutant showing leftward heart looping at E9.5. The embryo midline is outlined in blue. (**B** and **C**) Imaging of the same individual at E18.5, showing abnormal superoinferior ventricle position in the thoracic cavity, based on the orientation of the interventricular septum [(B), red line]. The explanted heart is shown in an anterior 3D view (c1). (c2) Anterior frontal section showing the discordant connection between the anatomic right ventricle (RV) and ascending aorta (Ao). (c3) Posterior frontal section showing the D-LOOP configuration and RV hypoplasia. (**D**) Sankey plot showing the direct association between looping class at E9.5 and revertant trajectory at E18.5 by longitudinal analysis. (**E**) Pairwise associations between a revertant trajectory and the indicated E18.5 anatomical features, ordered by color-coded *P* values. (**F** and **G**) Distribution of ventricle position, based on the orientation of the interventricular septum (IVS) (F), and of ventricle hypoplasia (G) in congruent and revertant *Nodal* mutants at E18.5. (**H**) Scatter plot of samples projected onto principal dimensions from a multiple correspondence analysis of 11 parameters in E18.5 *Nodal* mutant hearts with D-LOOP (*n* = 29, see table S4), color coded for the congruent or revertant trajectory. (**I**) Supervised classification of revertants, rightward congruents and leftward congruents with a random forest model on 14 parameters at E18.5 (*n* = 40, see table S4), identifies three main phenotypic signatures according to their importance into the model. (**J**) Graphical abstract highlighting two asymmetric morphogenesis steps. Variations in the direction of these processes in *Nodal* mutants result in three developmental trajectories, which stratify cardiac defects in heterotaxy at birth. A/P, anteroposterior; Hypo, hypoplastic; L/R, left-right; LV, left ventricle; S/I, superoinferior; VSD, ventricular septal defect. Scale bars, 500 μm.

We focused on the new category of revertants that we have tracked longitudinally for undergoing a step of heart remodeling after embryonic looping. Most of them (11 of 12) looped leftward at E9.5 and reverted as a D-LOOP at E18.5 (Figs. 2C and 6, A to C), whereas one sample looped rightward at E9.5 and reverted in L-LOOP at E18.5 (Fig. 2C). Revertants are thus mainly observed in looping classes 1 and 3, representing overall 61% of embryos that had looped leftward (Fig. 6D and table S4). Looping class 4 had no revertant. Then, we looked at the structural defects of revertants and found that they were significantly enriched in abnormal orientation of the interventricular septum (*P* = 0.02) and in ventricle hypoplasia (*P* = 0.04) (Fig. 6, E to G). MCA on 11 phenotypic features at E18.5 was able to segregate much of the revertant from the congruent D-LOOP (Fig. 6H). A supervised classification with a random forest model was able to correctly predict revertants, rightward congruents, and leftward congruents for 78% of samples, which is significantly better (*P* = 0 for a permutation test with 5000 permutations) compared to random classification (38% in average). Apart from ventricle laterality, which defines revertants, and its topological association with the malposition of the great arteries, the variables that were most important to determine revertants or congruents are the orientation of the interventricular septum and ventricle hypoplasia (Fig. 6I). Revertants thus appear as an important category able to stratify heterotaxy with right isomerism, based on developmental mechanisms. Despite the rescue of ventricle laterality, revertants are abnormal D-LOOP, keeping a signature of their abnormal trajectory in terms of ventricle malposition and hypoplasia (Fig. 6J).

### Characteristics of revertant heart anatomy are also detectable in patients

**Fig. 7.**
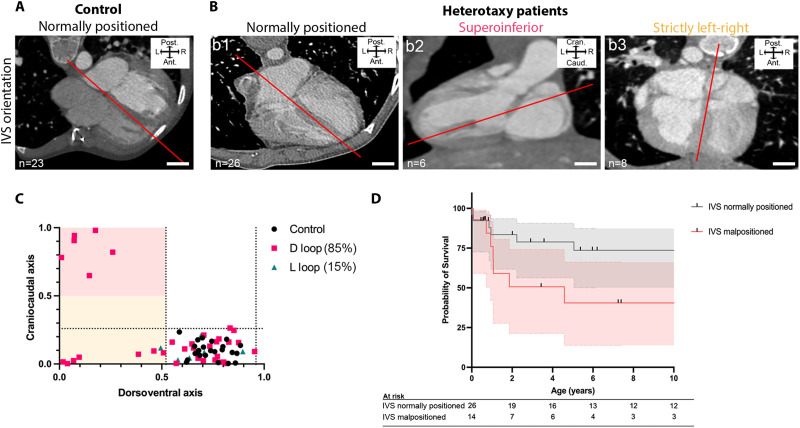
Ventricle position in patients with heterotaxy and right isomerism of bronchi. (**A** to **B**) CT scans of human hearts, from controls (A) and patients with heterotaxy and right isomerism of bronchi (B). The orientation of the interventricular septum (IVS) is indicated in red, from which the position of ventricles within the thoracic cavity is extracted. (**C**) Quantification, based on the craniocaudal and dorsoventral components of the vector perpendicular to the IVS plane, showing the distribution of ventricle position in controls (black dots) and heterotaxy patients with D-LOOP (pink squares) and L-LOOP (blue triangles). The dotted black lines correspond to the 99% distribution interval of the control distribution. Cases in the region highlighted in red have abnormal superoinferior ventricles and in yellow abnormal strictly left-right ventricles. (**D**) Survival curve of patients (D-LOOP and L-LOOP), comparing hearts with normally and abnormally positioned ventricles and their 95% confidence interval (*P* = 0.06, log-rank test, *n* = 40). Ant, anterior; cran, cranial; caud, caudal; L, left; Post, posterior; R, right. Scale bars, 2 cm (A), 1 cm (b1 and b3), and 0.5 cm (b2). See also tables S5, S6, and S8.

To assess whether reversion of ventricle laterality could also occur in humans, we interrogated a cohort of human patients with heterotaxy and right isomerism of the bronchi. Among 40 patients (Table S5), we detected a deficit in L-LOOP (15%), very similar to mouse *Nodal* mutants, which does not align with the randomization of embryonic heart looping direction (Fig. 2). The orientation of the interventricular septum was quantified in patient CT scans using the same approach as in the mouse (Fig. 7, A to C). Compared to control patients, we identified a number of heterotaxy patients who had abnormal D-LOOP with ventricle malposition: 6 were found with superoinferior ventricles and 7 with strictly left-right ventricles. Thus, ventricle anatomy in patients with heterotaxy and right isomerism is consistent with the existence of revertants. Following of patients (D-LOOP and L-LOOP) indicates that the group with ventricle malposition tends to have a twice higher mortality compared to patients with normal ventricle position (*P* = 0.06) (Fig. 7D and table S6). In the context of a small cohort, this notable effect size was not significant. Plasticity of ventricle position, which deviates from the normal developmental trajectory, thus appears as a factor worth to be taken into account for patients with heterotaxy and right isomerism of bronchi.

## DISCUSSION

We have performed a longitudinal analysis of heterotaxy, which uncovers the fate of variations in the embryonic heart loop shape in *Nodal* mutants. We find that the mature heart structure is not fully predicted by the embryonic heart shape, showing that *Nodal* can continue to modulate heart morphogenesis after heart looping. In particular, ventricle laterality is not set by the direction of heart looping. This unexpected level of plasticity reveals an extra step of asymmetric heart morphogenesis, required to maintain ventricle position. We show that it corresponds to a process of morphological remodeling of the heart at E13.5 rather than molecular reprogramming of the ventricles. Overall, we provide original insights into the embryological mechanisms of heterotaxy, which involve several steps of asymmetry beyond early symmetry breaking in the left-right organizer. Stratification of the phenotypic spectrum of heart defects in heterotaxy is better achieved by considering the direction congruence or reversal of the two steps of heart looping and remodeling. Plasticity in ventricle laterality is associated with a higher occurrence of ventricle malposition and hypoplasia. In patients, anatomical features consistent with revertants are potentially associated with poorer prognosis.

NODAL signaling is a major determinant of asymmetry, which provides long-term imprint. It becomes left-sided in the node (*28*) and is specifically transmitted to the left lateral plate mesoderm (*29*, *30*). It is transiently active in heart precursors (E8.5c-d), which later contribute to the arterial and venous poles of the heart tube at E9.5, but not to the ventricles (*14*). We show here that *Nodal* is not re-expressed within the heart after E9.5. Atria emerge on the left and right sides, as a bifurcation of the heart tube (*13*), and the left atrium is specifically colonized by precursor cells that have expressed *Nodal* (*14*). Atrium identity, but not position, is impaired in *Nodal* mutants, as seen by atrial isomerism. This reflects the nonactivation of the transcription factor PITX2, which is required for left atrial identity (*31*, *32*). This is exactly the opposite of ventricles, which are blind to NODAL signaling. Consistently, we did not detect ventricular reprogramming in *Nodal* mutants by genetic tracing experiments and molecular analyses. The molecular identity of left/right ventricles was also shown to be independent of left-right embryo patterning in *Dnah11 (iv)* mutants (*33*, *34*). Both ventricles are specified early in the straight tube as medial structures, with a craniocaudal relative position (*13*, *35*). It is thus the position, rather than the identity of ventricles, which depends on left-right patterning. During normal development, ventricles acquire their left/right position during the process of heart looping (*13*), which is modulated by NODAL signaling (*14*). We now show that a further step of heart remodeling postlooping, between E12.5 and E13.5, is required to maintain ventricle position. This remodeling appears sensitive to NODAL signaling, because it may be abnormal in *Nodal* mutants. At E9.5, when heart looping is complete, the heart tube is not fully elongated (*36*, *37*) and cardiac precursors in the dorsal pericardial wall have expressed *Nodal* on the left side (*14*). Thus, it is possible that ingression of precursors cells after heart looping continues to modulate heart shape based on their left/right patterning. In the case of heart looping, we showed that NODAL does not initiate asymmetry, but rather plays a biasing role to orient asymmetries (rightward or leftward), as well as an amplifier role of asymmetry intensity at the tube poles (*14*, *38*). Here, because reversal of ventricle laterality partially affects samples, this may reflect the biasing role of NODAL. The fact that revertants are enriched in malposition of the ventricles may involve the amplifier role of NODAL. Our analysis of *Nodal* mutants overall show that NODAL signaling can have long-term effect, modulating morphogenesis after it has been turned off. What are the relays of NODAL signaling is still incompletely understood. The transcription factor PITX2 is one of them, regulating atrial identity and heart loop shape, but not its direction (*14*, *17*, *39*, *40*).

Asymmetric morphogenesis of the heart integrates several steps, which may be uncoupled in mutant conditions. The growing heart tube deforms by a process of buckling, which is independent of NODAL signaling (*13*, *14*). Early asymmetries at the heart tube poles, modulated by NODAL signaling, orient buckling to generate a helical shape. This includes a rightward rotation of the arterial pole, asymmetric cell ingression at the venous pole, and a leftward displacement of the venous pole (*13*). In *Nodal* mutants, the shape of the heart loop is always abnormal (*14*), which potentially reflects the full penetrance of some congenital heart defects. The reduction in arterial pole rotation at E8.5f (*14*) is in keeping with parallel, malposed great arteries and defective ventriculo-arterial connections, whereas impaired displacement of the venous pole at E9.5 (*14*) may be at the origin of complete atrioventricular septal defects. Our longitudinal analysis now shows that the shape of heart looping in *Nodal* mutants is a partial predictor of the emergence of other congenital heart defects, given variations in the fate of a specific heart loop class. Whereas ventricle position was considered to be set during heart looping, we uncover that ventricle position needs to be maintained after heart looping. One can only speculate about the mechanisms of heart remodeling after heart looping, which underlies plasticity in ventricle position. Rotation of the outflow tract continues to manifest after heart looping, resulting in spiraling of the great arteries (*41*). It is possible that a change in the direction of outflow tract rotation could trigger reversion. Another asymmetry is detected between the pulmonary and aortic roots, when the plane of the aortic valve is reoriented at E12.5 at an angle to that of the pulmonary valve, facilitating the connection between the left ventricle and aorta (*42*). In *Nodal* mutants, because great arteries are always parallel and the aorta never correctly connects to the left ventricle, this mechanism is likely to be impaired with full penetrance. *Nodal*-expressing cells contribute to the outflow tract (*14*) and its downstream target *Pitx2* is required for the spiraling of the great vessels (*41*). The heart tube continues to elongate after heart looping. In a model of crisscross heart, we had shown that this is important to maintain ventricle position after heart looping (*37*). Growth arrest of the outflow tract in *Greb1l* mutants was proposed to generate mechanical tensions released by an ectopic rotation of the atrioventricular canal. Whether this process is involved in revertant *Nodal* mutants remains an open question. The overall mechanism controlling ventricle position integrates left-right patterning, heart tube growth, and mechanical constraints before and after heart looping. In the gut as well, several steps of asymmetry, beyond the initial symmetry breaking event, have been identified, to control its counterclockwise rotation. Early *Nodal* expression in the left mesoderm is followed by later *Bmp4*/*Pitx2* asymmetry in the dorsal mesentery amplified by mechanical feedback (*43*). After symmetry breaking in the left-right organizer, asymmetric organogenesis integrates further morphogenetic processes.

Even if the genetic origin of heterotaxy is known in 20% of cases (*2*, *4*, *44*), why it encompasses a broad phenotypic spectrum has remained poorly understood. Several organs can be affected, with variable association between specific defects and variable coordination of their laterality. In patients, congenital heart defects are the most important to determine prognosis. Our observations of more frequent D-LOOP than L-LOOP in mouse *Nodal* mutants and patients with heterotaxy has also been observed in human heart specimens (*45*). In *Nodal* mutants, we find that leftward looping does not lead to situs inversus totalis. It is instead associated with L-LOOP or abnormal D-LOOP that are enriched in ventricle malposition and hypoplasia. Our longitudinal analysis thus questions the clinical nomenclature of D-LOOP and L-LOOP, by invalidating the underlying embryological assumption. Whereas the predominant framework of symmetry breaking in the left-right organizer proposed a limited number of phenotypic outputs, situs solitus, situs inversus, right isomerism, and left isomerism (*46*), the identification of several steps of asymmetry afterward now provides better insights into the phenotypic heterogeneity of heterotaxy (*3*). We show that taking into account two steps of asymmetry, looping at E9.5 and remodeling at E13.5, better stratifies heterotaxy with right isomerism (Fig. 6J). Consistent observations in patients of a deficit of L-LOOP and the existence of abnormal D-LOOP suggest that plasticity of ventricle positions is conserved in humans. In addition, decreased survival trend of patients with abnormal D-LOOP suggests that ventricle position is an important factor to consider in future clinical studies of heterotaxy. Knowledge of the developmental trajectory of congenital heart defects provides insights into the origin of anatomical variations, which have an important impact for patient management and prognosis.

### Limitations of the study

Our study identifies a process of heart remodeling, which permits us to stratify the phenotypic spectrum of heterotaxy. We have used here a single model, *Nodal* mutants, which display a specific type of heterotaxy with right isomerism of the airways. Similar analyses in alternative models of heterotaxy, hitting different genetic factors, or targeting another part of the phenotypic spectrum such as left isomerism, will be interesting complements. Yet, this remains challenging in models in which heterotaxy has a low penetrance. We have generated a unique cohort from which the correspondence between the heart structure at E9.5 and E18.5 can be analyzed. We have identified E13.5 as a remodeling stage. However, the underlying mechanisms are still elusive. We have obtained encouraging results in a cohort of patients, showing anatomic similarities with the mouse model and suggesting that abnormal D-LOOP with malposition of the ventricles is a parameter worth taking into consideration. Demonstration that such a different developmental trajectory is a risk factor for patients is challenging, because of the difficulty to generate a large cohort of patients with a rare defect.

## MATERIALS AND METHODS

### Experimental design

This study was designed with the primary aim to stratify heterotaxy by generating a longitudinal cohort and analyzing the fate of specific configurations of embryonic heart looping. A secondary objective was to investigate whether observations in the mouse are consistent with that in patients.

### Animal model

*Nodal^null/+^; Hoxb1^Cre/+^* males (*14*) were maintained in a mixed genetic background and crossed to *Nodal^flox/flox^*; *R26R^LacZ/LacZ^* females (*47*) to generate *Nodal* conditional mutants. These mutants all display a normal embryonic turning. E0.5 was defined as noon on the day of vaginal plug detection. Primers used for genotyping are provided in table S7. Animals were housed in individually ventilated cages containing tube shelters and nesting material, maintained at 21°C and 50% humidity, under a 12-hour light/dark cycle, with food and water ad libitum, in the Laboratory of Animal Experimentation and Transgenesis of the SFR Necker, Imagine Campus, Paris. Animal procedures were approved by the ethical committees of the Institut Pasteur, Université Paris Cité and the French Ministry of Research (#18049-201707201335745 v9, #31733 2020120811176379_v6, and dha230022).

### Micro-ultrasound imaging and analysis

The longitudinal cohort was imaged as described in (*24*), from 21 litters. E9.5 embryos were imaged in utero noninvasively, transabdominally, using the Ultra High-Frequency Imaging Platform Vevo2100 (Visualsonics) with a 50-MHz probe (MS-700). Fast scans of the uterine horns and of each embryo were acquired to identify the embryo position in the uterus (table S3) and check its viability. A 3D + t scan of each embryo was then acquired across the deciduum. The dataset comprises 94 images with an axial and lateral resolution of 30 and 50 μm, respectively. The motor has a step size of 32 μm. 3D stack of the micro-ultrasound was processed using the Volume Viewer plugin on Fiji (ImageJ) as well as the DIVA (Data Integration and Visualization in Augmented and Virtual Environments) software (*48*) (https://github.com/DecBayComp/VoxelLearning). 3D coordinates were positioned at the level of the head, tail, right, and left of the embryo, to define anteroposterior and left-right axes, respectively. Four additional points were positioned in the center of each ventricular and atrial chambers (fig. S2B). The final plot of heart chamber position was generated using an in-house Matlab code (source code S1). In agreement with our previous observations (*14*), the four classes of heart looping (fig. S1A) and the direction of heart looping (Fig. 2, A and C) are randomly distributed according to a uniform distribution. Figure panels correspond to optical sections in the plane which is most appropriate, i.e., not necessarily in the plane of imaging.

### E9.5 heart phenotyping

The class of embryonic heart looping in *Nodal* mutants is defined on the basis of the position of the ventricles, the outflow tract and the inner curvature. In class 1, looping is oriented leftward, ventricles are inversely lateralized, and the inner curvature is caudal. In class 2, looping is oriented rightward and both ventricles lie on the right side. In class 3, looping is oriented leftward, the right ventricle lies on the left side, the left ventricle is medial, and the inner curvature is cranial. In class 4, looping is oriented rightward and ventricles are normally lateralized. Note that dissection was later (evening versus morning) compared to Desgrange *et al.* (*14*). In a control experiment of ultrasound imaging and phenotyping, eight litters of *Nodal* mutants and control littermates were dissected immediately after in utero ultrasound imaging at E9.5 (fig. S2 and movie S1), to control the classification of heart looping made from ultrasound images. Bright-field images were acquired with a Zeiss AxioCamICc5 Camera and a Zeiss StereoDiscovery V20 stereomicroscope with a Plan Apo 1.0× objective. Mutant samples from this ground truth cohort were imaged in 3D by HREM, their heart tube segmented with Napari, and a semi-automatic self-supervised learning procedure, which will be published later.

### Embryo and fetus collection

E18.5 fetuses were recovered with their positions in the uterine horns carefully monitored (table S3). Fetuses were euthanized by decapitation. The body was subsequently immerged in HBSS at 37°C for 5 min to remove blood, then in cardioplegia solution for 5 min to arrest the heart in diastole. For the first half of the cohort, we used the cardioplegia solution 250 mM KCl; for the second half, we used an improved solution of 110 mM NaCl, 16 mM KCl,16 mM MgCl_2_, 1.5 mM Cacl_2_, and 10 mM NaHCO_3_ (table S2). The body was fixed in 4% paraformaldehyde 24 hours at 4°C. Embryos and fetuses at E9.5, E10.5, E12.5, E13.5, and E14.5 were similarly collected in independent kinetic cohorts. Both male and female fetuses were collected and used blindly for experiments. All samples were genotyped by polymerase chain reaction (PCR), using primers listed in table S7.

### Micro-CT

The thoracic skin was removed to improve penetration of the contrast agent; the left arm was removed, as a landmark of the fetus left side. Samples were stained in 100% Lugol over 72 hours (*24*). Images of the thorax and abdomen were acquired on a Micro-Computed Tomography Quantum FX (Perkin Elmer), within a field of exposure of 10 mm diameter (movie S2). The dataset, comprising 512 images of 20 × 20 × 20 μm *x*-*y*-*z* resolution for each sample, was analyzed using Imaris software (Bitplane). Figure panels correspond to optical sections in the plane that is most appropriate, i.e., not necessarily in the plane of imaging.

### High-resolution episcopic microscopy

Embryos (at E9.5, E10.5, and E13.5) or explanted hearts (at E12.5, E14.5, and E18.5) were embedded in a methacrylate resin (JB4) containing eosin and acridine orange as contrast agents. One or two channel images of the surface of the resin block were acquired, using the optical high-resolution episcopic microscope and a 1× Apo objective, repeatedly after removal of 2.6-μm-thick sections: The tissue architecture was imaged with a green fluorescent protein filter (movie S3) and, where applicable, the staining of β-galactosidase precipitates with a red fluorescent protein filter. The dataset comprises 800 to 2200 images of 1.7 to 5 μm resolution in *x* and *y*. Occasionally, one slice may be lost or overexposed, creating a minor variation in the overall 3D stack. Icy and Fiji softwares were used to crop or scale the datasets and merge channels. 3D reconstructions were performed with the Imaris Software (Bitplane). Figure panels correspond to optical sections in the plane that is most appropriate, i.e., not necessarily in the plane of imaging.

### E18.5 fetus phenotyping

Cardiac anatomy was phenotyped twice by a biologist and a cardiac pediatrician based on 3D images acquired by micro-CT and HREM, using the segmental approach (*23*) and IPCCC ICD-11 code. The position of the heart apex corresponds to that of the apex of the left ventricle within the thoracic cavity. The anatomic right and left ventricles are defined on the basis of the presence and absence of a septal attachment of the atrioventricular valve, respectively (Fig. 2B). The laterality of ventricles reflects in most cases the congruent (D-LOOP) or not (L-LOOP) position of the anatomic right ventricle on the right side of the left ventricle. In case of ventricle malposition (superoinferior or anteroposterior), we additionally used the principle of handedness of the tripartite ventricle as defined by Van Praagh *et al.* (*25*). The position of ventricles based on the orientation of the interventricular septum was quantified in micro-CT scans using reference axes of the fetus (Supplementary Text and fig. S3, A and B). Malposition of the great arteries was diagnosed in a transverse plane, based on the position of the aortic valve relative to the pulmonary valve. The ventriculo-arterial connection reflects the abnormal position of the great arteries: double outlet right ventricle, when more than half of both arterial trunks arise from the anatomic right ventricle, transposition of the great arteries when the aorta arises from the anatomic right ventricle, and the pulmonary trunk from the anatomic left ventricle. The orientation of the aortic arch, the entry point of caval veins, and pulmonary vein collector relative to the midline (dorsoventral plane crossing the spinal cord and sternum) was diagnosed in micro-CT scans. Ventricle hypoplasia was quantified on the basis of HREM image segmentations and the ratio between the right and left ventricle outer volume (Supplementary Text and fig. S3, C to F). Hypoplasia of the arterial trunks was quantified on the basis of HREM image segmentations and the ratio between the aortic and pulmonary valve diameter (Supplementary Text and fig. S3, G to J). The anatomic right and left atria are defined on the basis of the presence and absence of pectinate muscles in the posterior wall of the atrial chamber, respectively (fig. S1b4). The anatomic right and left lungs are defined based on the number of lobes (four on the right and one on the left, fig. S1b2). The anatomic right and left bronchi are defined on the basis of their subdivision (early on the right and late one on the left, fig. S1b2). Heterotaxy was defined according to the criteria of Lin *et al.* (*3*). Individual phenotypes are shown in table S4.

### Heart phenotyping at E10.5 to E14.5

The right ventricle was defined on the basis of its continuity with the proximal outflow tract at E10.5. From E12.5 to E13.5, before the formation of the atrioventricular valves, the right ventricle identity was based on its protruding infundibulum, which derives from the proximal outflow tract (*21*, *49*). At E14.5 and E18.5, the presence of the infundibulum correlates with other anatomical features specific of the right ventricle, i.e., a septal attachment of the atrioventricular valve. The rightward heart configuration corresponds to a right-sided proximal outflow tract or infundibulum compared to the left ventricle, devoid of infundibulum.

### β-Galactosidase staining

A separate cohort of 21 *Nodal^flox/null^*;*Hoxb1^Cre/+^;R26R^lacZ/+^* and 12 *Nodal^flox/+^*;*Hoxb1^Cre/+^;R26R^lacZ/+^* littermate controls was collected at E18.5. Isolated hearts were fixed in 4% paraformaldehyde, 5 mM EGTA, and 2 mM MgCl_2_ for 30 min. They were permeabilized in 0.2% NP40, 2 mM MgCl_2_, 0.1% sodium deoxycholate 30 min, and stained whole-mount 2h30 in Xgal solution at 37°C. Bright-field images were acquired with a Zeiss AxioCamICc5 Camera and a Zeiss StereoDiscovery V20 stereomicroscope with a Plan Apo 1.0× objective.

### RT-qPCR

Microdissected heart fields and heart tube at E8.5g-h or micro-dissected left and right ventricular walls, excluding the interventricular septum, at E18.5 were collected in cold phosphate-buffered saline. Ventricle laterality at E18.5 during such fast microdissection was determined on the basis of the position of the great arteries, which correlates with it (Fig. 4E) and the location of the infundibulum. The tissue was flash-frozen in liquid nitrogen. All samples were collected within 1 hour of euthanizing the mother. RNAs were extracted in TRIzol-chloroform and purified using RNeasy micro (at E8.5) or mini (at E18.5) kits (Qiagen). Reverse transcription was performed using a Reverse Transcription kit (QuantiTect, Qiagen). Quantitative PCR (qPCR) was carried out using the Quant Studio 3 PCR system and the FastStart Universal SYBR Green Master kit. mRNA expression levels were measured relatively to *Polr2b* and normalized with a reference cDNA sample (taken as a pool of four heart fields at E8.5g-h, or a pool of three right and three left ventricles at E18.5), using the standard ΔΔCt method. Ratio of paired right and left ventricles were computed. Primers are listed in table S7.

### Patient recruitment and cardiac imaging

Data were collected retrospectively after review of patient records (clinical characteristics and echocardiography). Among 505 patients with heterotaxy and control patients in the department of Pediatric Radiology in Necker-Enfants Malades Hospital, 138 were selected with available thoracic CT scans and 40 of them with right isomerism of the bronchi, similar to mouse *Nodal* mutants. Twenty-three control patients were selected with normal ventricle laterality (D-LOOP). Control patients had repaired simple transposition of the great arteries (CT scan indication for coronary arteries), aortic aneurysm, left pulmonary artery sling, or aortic arch anomalies. Patient CT examinations were done using a Lightspeed VCT 64-slices, GE Healthcare (coverage 4 cm of patient anatomy per rotation, gathering 64 slices at 0.625 mm) (*50*). During CT acquisitions, contrast (iomeron, 350 mg/ml, 2 ml/kg) was injected at a flow rate determined by body size and intravenous access size (from 1 to 3.5 ml/s) followed by a saline flush using a power injector. Image reconstruction was performed with a slice thickness of 0.625 mm, an increment of 0.625 mm, and the STANDARD reconstruction kernel. Iterative reconstruction was used with 60% ASIR. CT scans were completely anonymized, in accordance with the procedures of the APHP data protection office (no. 2024 0704182149). In patients, the anatomic right ventricle was distinguished from the anatomic left ventricle based on the presence of a moderator band, the more apical position of the tricuspid valve and its septal attachment. The cardiac phenotype of patients (table S5) was similar to that of mouse *Nodal* mutants, with high penetrance of complete atrioventricular septal defect (75%), malposition of the great arteries (93%) and abnormal ventriculo-arterial connections (93%), and a similar distribution of heart apex position (45% levocardia, 17% mesocardia, 38% dextrocardia) and aortic arch position (57% left, 43% right).

### Quantification of ventricle position based on the orientation of the interventricular septum

Analysis was performed on the 3D reconstruction of patient heart CT scans, using Imaris (Bitplane) and Matlab softwares. Analysis of ventricle position is based on the orientation of the interventricular septum, as described previously (*37*). The plane of the interventricular septum was identified anatomically and placed in 3D using an Oblique slicer. Three points were marked on the surface of the plane to define two vectors. Then, the plane was defined by the normalized cross product of the two vectors, i.e., the orientation of the vector perpendicular to the plane of the interventricular septum. The reference craniocaudal axis was defined along the spine. The reference dorsoventral axis was taken in a single transversal plane, with two points on the spine. It was then readjusted at 90° with the spine using an in-house Matlab code (https://doi.org/10.5281/zenodo.8265324). Both axes were thus marked by two points defining a vector, which was normalized. The reference left-right axis was obtained by the cross product of the craniocaudal and dorsoventral vectors. Each 3D coordinates, reflecting ventricle position, was obtained by the dot product of the normalized interventricular septum vector with each normalized reference axis vector (craniocaudal, dorsoventral, and left-right) using a Matlab code (source code S2), so that their Euclidean norm equals one. The diagnosis of strictly left-right and superoinferior ventricles was based on the vector component with the highest value, i.e., the left-right and dorsoventral component, respectively, outside the 99% distribution interval of control samples (Fig. 7C).

### Statistical analyses

Sample sizes were computed using power analysis to ensure the statistical detection of effect sizes with medium to large effects [Cohen’s *d* ≥ 0.5 for *t* tests, Cohen’s *f* ≥ 0.25 for analyses of variance (ANOVAs), and Cohen’s *w* ≥ 0.3 for χ^2^ and Fisher tests] with a type I error rate of 0.05 and 80% power. All sample numbers (*n*) indicated in the text refer to biological replicates, i.e., different embryos or different cells. In the experimental design, litter reflects an independent replicate of the experiment, with at least five litters per experiment. Group allocation was based on PCR genotyping. Investigators were blinded to allocation during imaging and phenotype analysis, but not during quantifications. Statistical tests and *P* values are described in figure legends and table S8. *P* values less than 0.05 were considered statistically significant. A χ^2^ test was used to compare percentage distributions. A Fischer exact test with adjusted *P* value for multiple testing using Benjamini-Hochberg correction was used to test pairwise associations. A Student *t* test was used when comparing two means and an ANOVA when comparing four groups. Patient survival plots were made according to the Kaplan-Meier method and survival functions were compared using a log-rank test. Sample exclusion in some analyses of the longitudinal cohort is specified in table S4. In the genetic tracing cohort, four samples (one control, one L-LOOP, and two D-LOOP), which showed no X-gal staining and one damaged sample were considered as outliers and removed from the analysis. In the kinetic cohorts, one degenerated sample at E12.5 was excluded from the analysis.

### Multiple correspondence analysis

MCA is a dimension reduction method, an extension of the well-known principal components analysis specifically dedicated to the analysis of qualitative data. It is implemented in FactoMineR R package (*51*). Eleven variables in the longitudinal cohort at E18.5 (orange in table S4) were used to compute the principal dimensions. Potential batch effects were tested for litter and cardioplegia on the MCA. Six samples were excluded from the MCA due to uncertain diagnosis: 2 samples had unknown looping class (because ultrasound acquisition was not in 3D), 1 sample had unknown ventricle laterality (because of indistinguishable atrioventricular valves after incomplete cardioplegia), and 3 samples had ambiguous situs of auricular appendages. All the results can be found in the accompanying web interface. MCA interpretation is mainly based on individual projection on principal components. These graphs are accompanied by quantitative quality measure tables such as *v* tests and variable contributions, which allow to identify associations between principal components and levels of the variables. For each principal component, contributions denote values between 0 and 100%, indicating how much a level of a variable is associated to the aforementioned component. *v* tests also reflect associations between principal components and variables but are more convenient for interpretation as they can be assimilated to *Z* scores, meaning that a threshold of 2 in absolute value indicates strong associations between a principal component and a variable level. More methodological details about MCA were described by Greenacre and Blasius (*52*).

### Pairwise associations between parameters

Associations between parameters of the longitudinal cohort were tested. To test the pairwise association between a given parameter (Figs. 1F and 6E) and a combination of multiple parameters at E18.5, we used a Fisher exact test with *P* values adjusted on multiple testing using Benjamini-Hochberg method. Visualization of associations was represented in Sankey plots. Both statistical tests and visualizations were performed using R. All the results can be found in the accompanying web interface.

### Supervised classification models

The ability of 14 parameters at E18.5 (orange and pink in table S4) to predict either the associated E9.5 looping class or the revertant status was assessed using random forests (*n* = 40). We used the implementation of this prediction method available in ranger R package (*53*). Both parameters “mtry” and “number of trees” were estimated using leave-one-out cross-validation (LOO-CV), choosing the combinations of parameters leading to the lower predictor error on the testing set. Variable importance was computed using Gini index. In the parameter estimation step, when several models reached the same performance, we averaged the corresponding variables importance to get a consensual ranking of the predictors. Prediction accuracy (proportion of correctly predicted samples) was also estimated using an independent LOO-CV procedure. To assess the significance of the prediction score (% of correctly classified samples), a permutation test was performed. At each step of the LOO-CV, the response of the testing sample was randomly picked among the responses of the training set. Repeating this process 5000 times allowed to compute the distribution of the prediction accuracy of a random prediction, which can be interpreted as the distribution under the null hypothesis “no prediction signal into the covariates.” A *P* value is deduced using empirical quantiles of this distribution.

## Supplementary Materials

### The PDF file includes:

Supplementary Text Figs. S1 to S4 Legends for tables S1 to S8 Legends for movies S1 to S3 Source codes S1 and S2 References
