# Supplementary material for: Plasticity of ventricle position after heart looping in heterotaxy with right isomerism
Source: Sci Adv. 2025 Sep 19;11(38):eads8192. doi: 10.1126/sciadv.ads8192 (PMC12448140; doi:10.1126/sciadv.ads8192)
Supplement: Supplementary file 1 — Supplementary Text Figs. S1 to S4 Legends for tables S1 to S8 Legends for movies S1 to S3 Source codes S1 and S2 References [file sciadv.ads8192_sm.pdf]

Science Advances
AAAS

Supplementary Materials for

Plasticity of ventricle position after heart looping in heterotaxy with right isomerism

Audrey Desgrange *et al.*

Corresponding author: Sigolène M. Meilhac, sigolene.meilhac@institutimagine.org

Sci. Adv. **11**, eads8192 (2025)
DOI: 10.1126/sciadv.ads8192

The PDF file includes:

Supplementary Text
Figs. S1 to S4
Legends for tables S1 to S8
Legends for movies S1 to S3
Source codes S1 and S2
References

Other Supplementary Material for this manuscript includes the following:

Tables S1 to S8
Movies S1 to S3

Supplementary Text

Supplementary material and methods

Clustering of looping classes by Principal Component Analysis (PCA) of E9.5 embryos

In a control experiment, in which control and mutant E9.5 embryos were dissected immediately after ultrasound imaging, quantitative parameters of heart shape were extracted in 16 mutant embryos, as in Desgrange et al (*14*) and computed in Matlab where applicable. The origin for the 3D reference axis was set at the distal outflow tract (exit of the tube). 26 variables per embryo were used for the PCA analysis: 3D coordinates of 7 reference points (centroids of outflow tract volume, right ventricle volume, interventricular polygon, left ventricle volume, atrioventricular canal and left atrium volume, right atrium volume, position of the venous pole taken as the bifurcation point of the tube into the atria), 4 angles (3 between 3 consecutive reference points, and the orientation of the left and right ventricles relative to the notochord), tube length. An unscaled PCA was performed using the FactoMineR R package. A hierarchical clustering on the 16 *Nodal* mutants was performed on the three first principal components using the Euclidean distance and the Ward.D2 aggregation criterion. The PCA validates 100% accuracy of looping direction determination by ultrasound imaging and excellent looping class allocation, with a 5% error (Fig. S2).

Quantification of ventricle position based on the orientation of the interventricular septum

Analysis was performed on the micro-CT scans of mouse E18.5 fetuses, using the same approach as in patient CT-scan described in the main text. The reference dorso-ventral axis was taken in a single transversal plane, with one point on the spine and one point on the most ventral point of the body (at the level of the sternum). The 3 vector components (cranio-caudal, dorso-ventral, left-right) are normalised so that their Euclidean norm equals one. The diagnosis of strictly left-right, supero-inferior and antero-posterior ventricles was based on the vector component with the highest value, i.e. the left-right, cranio-caudal and dorso-ventral component, respectively, outside the 95% distribution interval of control samples (Fig. S3B).

Quantification of ventricle hypoplasia based on their volumes

Analysis was performed on the 3D HREM images of E18.5 hearts. 3D outer volume of the right (RV) and left (LV) ventricle was manually segmented and computed using the Surface tool of Imaris (Bitplane). We used the basal limit of myocardium as a landmark and the middle of the septum to separate the left and right ventricles. In mutants, which have ventricular septal defects, the interventricular septum was prolonged along its main axis. Ventricle hypoplasia in *Nodal* mutants was diagnosed based on the RV/LV ratio of ventricle outer volumes outside the 95% distribution interval of the control samples (Fig. S3F). Measurements were also performed on the inner volume of ventricles. However, we noticed that whole mount cardioplegia of fetuses with 250mM KCl was not always sufficient to fully relax the myocardium, corresponding to cases of abnormally small inner ventricular volumes in both controls and mutants. No sign of myocardium hypertrophy was detected in the cohort of cardioplegia with 110mM NaCl, 16mM KCl,16mM $MgCl_2$, 1.5mM $CaCl_2$, 10mM $NaHCO_3$. In this case of efficient cardioplegia, the ratio of RV/LV yielded the same conclusion using the outer or inner volumes.

Quantification of arterial trunk hypoplasia based on their diameter

Analysis was performed on the 3D HREM images of E18.5 hearts. The longest diameter of the ascending aorta (Ao) and pulmonary trunk (PT) was measured at the level of the arterial valves, after 3D segmentation of the arterial trunks using an oblique slicer on Imaris (Bitplane). Arterial trunk

hypoplasia in *Nodal* mutants corresponds to PT/Ao ratio of diameters outside the 95% distribution interval of the control samples (Fig. S3J).

Bioinformatics analyses of single cell RNA sequences

Data from Feng et al. (*54*) were downloaded following instructions of the publication. The analysis was reduced to cells from CD1 mouse hearts between E9.5 and E18, resulting in 20,000 cells in total, of which 11,300 are ventricular cardiomyocytes. Log-normalised expression levels were plotted according to cell types, dissected cardiac chambers and/or stages. P-values were computed with the Hurdle model implemented in the MAST R package (https://rglab.github.io/MAST/).

RNA *in situ* hybridization

RNAscope ISH was performed wholemount with Mutliplex Fluorescent v2 Assay (Advanced Cell Diagnostic, cat. no.323110) as described in (*14*). E8.5 wild-type embryos were fixed 24h in PFA at 4% and dehydratated in methanol 100%. mm-*Nodal*-C1 (Cat No. 436321) probe was used, together with Hoechst as a nuclear counterstain. Amplification steps were performed using the TSA cyanine5 amplification kit (Akoya Bioscience). Samples were then transferred in R2 CUBIC clearing reagents. Multi-channel 16-bit images were acquired with a Z.1 lightsheet microscope (Zeiss) and a 20X/1.0 objective.

Genetic tracing of *Nodal*

Embryos of the *Tg Nodal-ASE-lacZ* line (*55*) were collected at E9. The heart was arrested in diastole with 250mM KCL. Embryos were fixed in 4% PFA – 5mM EGTA – 2mM MgCl2 for 10min, permeabilised and stained with Xgal as described in the main text. 3D images were acquired by HREM with a x/y and z resolution of 1.43µm and 1.56µm, respectively.

Genetic tracing of *Hoxb1* at E9.5

Endogenous fluorescent embryos at E9.5 generated by crossing *Hoxb1*$^{Cre/+}$ (*56*) with *R26*^{mTmG} (Gt(ROSA)26Sortm4(ACTB-tdTomato,-EGFP)Luo) (*57*) reporter mice, were fixed 24h in PFA and counterstained with Hoechst as a nuclear marker. Samples were cleared in R2 CUBIC reagents. Multi-channel 16-bit images were acquired with a Z.1 lightsheet microscope (Zeiss) and a 20X/1.0 objective.

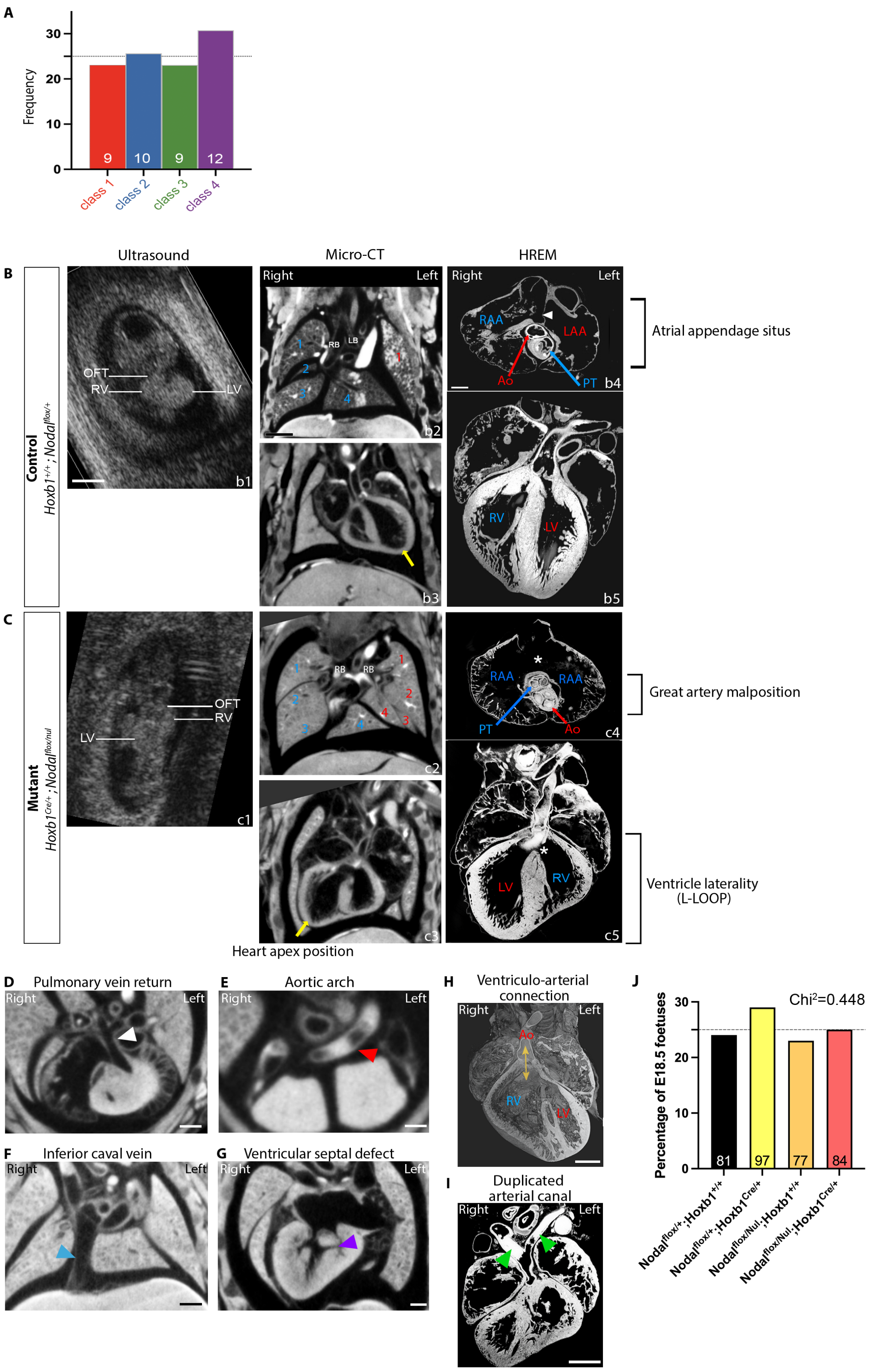
A
Frequency
30
20
10
0
9
10
9
12
class 1
class 2
class 3
class 4
Ultrasound
Micro-CT
HREM
B
Control
Hoxb1+/+ ; Nodalflox/+
OFT
RV
LV
b1
Right
Left
RB
LB
b2
b3
RAA
LAA
Ao
PT
b4
Atrial appendage situs
RV
LV
b5
C
Mutant
Hoxb1Cre/+ ; Nodalflox/nul
OFT
RV
LV
c1
RB
RB
c2
c3
Heart apex position
RAA
RAA
PT
Ao
c4
Great artery malposition
LV
RV
c5
Ventricle laterality
(L-LOOP)
D
Pulmonary vein return
E
Aortic arch
F
Inferior caval vein
G
Ventricular septal defect
H
Ventriculo-arterial
connection
Ao
RV
LV
I
Duplicated
arterial canal
J
Chi²=0.448
Percentage of E18.5 foetuses
30
20
10
0
81
97
77
84
Nodalflox/+;Hoxb1+/+
Nodalflox/+;Hoxb1Cre/+
Nodalflox/Nul;Hoxb1+/+
Nodalflox/Nul;Hoxb1Cre/+

Fig. S1. Multimodality imaging pipeline for the longitudinal analysis of heart defects in heterotaxy with right isomerism

(A) Heart looping class distribution observed in *Nodal* mutants imaged by ultrasound at E9.5. As expected from (*14*), the distribution did not deviate from an uniform distribution hypothesis (dotted line) (p=0.9, chi-square test, n as indicated). (B-C) Examples of control (B) and *Nodal* mutant (C) samples imaged with a multimodality imaging pipeline, including ultrasound imaging in utero at E9.5 (b1-c1), micro-CT (b2-c3) and HREM (b4-c5) imaging at E18.5. (b1-c1) Snapshot from the 3D+t ultrasound image, showing the position of the outflow tract (OFT), right (RV) and left (LV) ventricles. (b2-c3) Frontal sections of the thorax showing the situs of the lung lobes (coloured numbers) and bronchi (RB, LB) and the position of the heart apex (yellow arrow). In controls (b2) the right lung has 4 lobes (numbered in blue) and the left lung 1 lobe (numbered in red). In *Nodal* mutants (c2) 4 lobes are seen on the left and right, indicative of right isomerism. The heart apex position (yellow arrow) is in levocardia in controls (b3) and dextrocardia in this mutant sample (c3). (b4-c4) Transverse sections at the level of the arterial valves. In controls (b4), the right (blue, RAA) and left (red, LAA) atrial appendages have an asymmetric anatomy, and the ascending aorta (Ao) is posterior and right-sided relative to the pulmonary trunk (PT). The white arrowhead points to the interatrial septum. In the *Nodal* mutant shown (c4), isomerism of the right atrial appendages can be seen, absent atrial septation (asterisk) and transposition of the great arteries in L-malposition. (b5-c5) Frontal sections showing the anatomy of ventricles. In controls (b5), the right (RV, blue) and left (LV, red) ventricles are correctly lateralised (D-LOOP) and septated. In the *Nodal* mutant shown (c5), the anatomic right ventricle is abnormally on the left side (L-LOOP). Combined atrial (c4) and ventricular (c5, asterisk) septal defects are indicative of an atrioventricular canal. (D-E) Coronal sections from micro-CT images showing the insertion of the pulmonary vein collector in the left atrium (D, white arrowhead) and the left aortic cross (E, red arrowhead). (F-G) Transversal sections from micro-CT images showing the inferior caval vein insertion in the right atrium (F, blue arrowhead) and an additional ventricular septal defect (G, purple arrowhead). (H-I) Transversal sections of HREM 3D reconstruction showing a defective ventriculo-arterial connection (H, double outlet right ventricle) and a duplication of the arterial canal (I, green arrowheads). (J) Histogram showing the percentage of genotypes recovered in E18.5 litters of $Nodal^{null/+}$; $Hoxb1^{Cre/+}$ x $Nodal^{flox/flox}$ crosses. The observed frequency is not significantly different from the expected Mendelian ratio (dotted line) (p-value = 0.448, chi-squared test, n as indicated). Scale bars: 200µm (b1-c1 and H-I), 1000µm (b2-c3), 400µm (b4-c5), 500µm (D-G). See also Table S8.

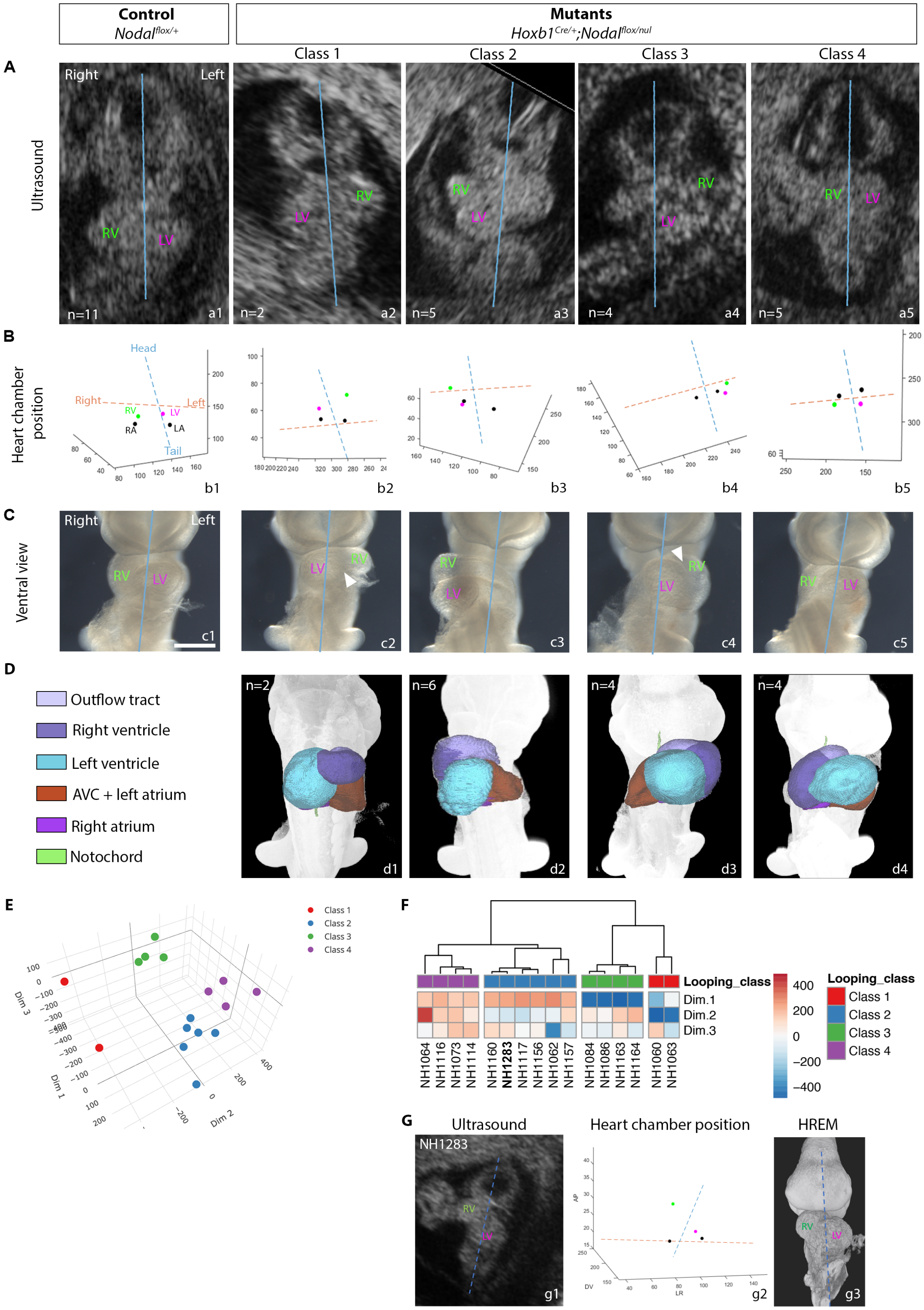

Control
Nodal flox/+
Mutants
Hoxb1 Cre/+;Nodal flox/nul
Class 1
Class 2
Class 3
Class 4
A
Ultrasound
Right
Left
RV
LV
n=11
a1
n=2
a2
n=5
a3
n=4
a4
n=5
a5
B
Heart chamber position
Head
Tail
RA
LA
b1
b2
b3
b4
b5
C
Ventral view
c1
c2
c3
c4
c5
D
Outflow tract
Right ventricle
Left ventricle
AVC + left atrium
Right atrium
Notochord
n=2
d1
n=6
d2
n=4
d3
n=4
d4
E
Class 1
Class 2
Class 3
Class 4
Dim 1
Dim 2
Dim 3
F
Looping_class
Dim.1
Dim.2
Dim.3
NH1064
NH1116
NH1073
NH1114
NH1160
NH1283
NH1117
NH1156
NH1062
NH1157
NH1084
NH1086
NH1163
NH1164
NH1060
NH1063
400
200
0
−200
−400
Looping_class
Class 1
Class 2
Class 3
Class 4
G
Ultrasound
Heart chamber position
HREM
NH1283
g1
AP
DV
LR
g2
g3

Fig. S2. Quantification of heart looping shape in a ground truth cohort at E9.5

Control experiment of ultrasound diagnosis at E9.5. Eight litters were imaged by in utero ultrasound imaging. 16 mutants and 11 controls were dissected immediately after. Paired images of the same sample at the different analytic steps are shown in columns. (A) Snapshot from the 3D+t ultrasound image of a control (a1) and *Nodal* mutants (a2-a5). The embryo midline is outlined in blue. (B) Analysis of 3D+t ultrasound images by virtual reality. The antero-posterior (blue dotted line) and left-right (orange dotted line) axes were marked, as well as the center of each heart chamber. This permits us to classify the heart loop shape, based on the left/right/medial position of the ventricles. In addition, the cranial/caudal inner curvature is extracted from the fast scans (Movie S1). (C) Brightfield images of samples after dissection (also presented in Fig. 1B), reflecting the in utero diagnosis. The cranial/caudal inner curvature is indicated by a white arrowhead. (D) 3D rendering of the embryo in a ventral view, after HREM imaging and segmentation of the heart. Cardiac regions are colour-coded as indicated. The notochord (green) is used as a reference axis to align samples. (E) Principal component analysis of 26 geometrical parameters characterising the segmented heart shape in the 16 mutants. (F) Corresponding unsupervised clustering tree of samples, supporting the classification of embryonic heart looping in *Nodal* mutants in four distinct shapes (Class 1-4). Looping direction diagnosed by ultrasound is confirmed in 100% cases, whereas the heart looping class is confirmed in 94% (15/16) mutants. (G) Illustration of sample NH1283, which had been incorrectly classified as Class 4 instead of Class 2 during ultrasound image analysis. This is because its left ventricle is more medial compared to other Class 2 mutants. Overall, ultrasound imaging correctly allocates looping classes with a 5 % error, which did not alter the performance of the supervised classification of E18.5 anatomic features, as prediction accuracy is similar using any of the two allocations. Ao, aorta; AVC, atrio-ventricular canal; LA, left atrium; LAA, left auricular appendage; LB, left bronchus; LV, left ventricle; OFT, outflow tract; PT, pulmonary trunk; RA, right atrium; RAA, right auricular appendage; RB, right bronchus, RV, right ventricle Scale bars: 200µm (a1 and E), 1000µm (a2), 400µm (a4). See also Table S8.

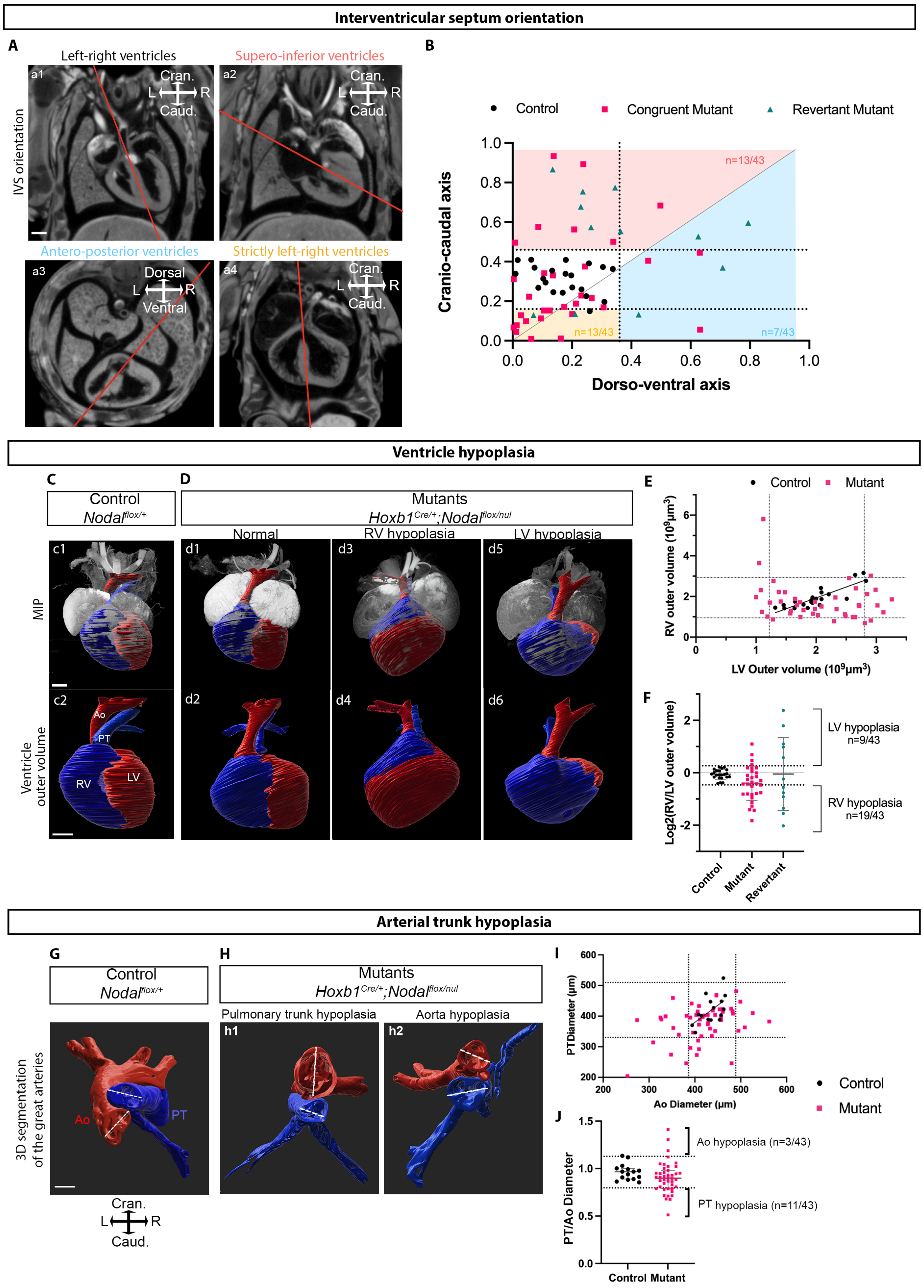

Interventricular septum orientation
A
Left-right ventricles
Supero-inferior ventricles
Antero-posterior ventricles
Strictly left-right ventricles
IVS orientation
a1
a2
a3
a4
Cran.
Caud.
Dorsal
Ventral
L
R
B
Control
Congruent Mutant
Revertant Mutant
Cranio-caudal axis
Dorso-ventral axis
n=13/43
n=13/43
n=7/43
Ventricle hypoplasia
C
Control
Nodal flox/+
D
Mutants
Hoxb1 Cre/+;Nodal flox/nul
Normal
RV hypoplasia
LV hypoplasia
MIP
Ventricle outer volume
c1
c2
d1
d2
d3
d4
d5
d6
Ao
PT
RV
LV
E
Control
Mutant
RV outer volume (10⁹µm³)
LV Outer volume (10⁹µm³)
F
Log2(RV/LV outer volume)
LV hypoplasia n=9/43
RV hypoplasia n=19/43
Control
Mutant
Revertant
Arterial trunk hypoplasia
G
Control
Nodal flox/+
H
Mutants
Hoxb1 Cre/+;Nodal flox/nul
Pulmonary trunk hypoplasia
Aorta hypoplasia
h1
h2
3D segmentation of the great arteries
Ao
PT
Cran.
Caud.
L
R
I
PTDiameter (µm)
Ao Diameter (µm)
Control
Mutant
J
PT/Ao Diameter
Ao hypoplasia (n=3/43)
PT hypoplasia (n=11/43)
Control
Mutant

Fig. S3. Quantification of interventricular septum orientation, ventricle hypoplasia and hypoplasia of arterial trunks

(A) Micro-CT images of the thoracic cavity at E18.5, showing examples of normal left-right (a1), abnormal supero-inferior (a2), antero-posterior (a3) or strictly left-right ventricle position, based on the orientation of the interventricular septum (red line). (B) Corresponding quantification, based on the cranio-caudal and dorso-ventral components of the vector perpendicular to the IVS plane, showing the distribution of ventricle position in controls (black dots) and *Nodal* mutants (pink squares and blue triangles). Mutants in the region highlighted in pink are defined as supero-inferior ventricles, in the blue region as antero-posterior ventricles and in the yellow region as strictly left-right ventricles. (C-D) HREM 3D images of hearts at E18.5 in control (C) and *Nodal* mutants (D). (c1, d1, d3, d5) 3D rendering seen in an anterior view, with the ascending aorta and left ventricle segmented in red and the pulmonary trunk and right ventricle in blue. (c2, d2, d4, d6) Focus on the segmented arterial trunks and ventricle outer volumes of the same hearts, showing balanced ventricle volumes (c2, d2), right ventricle hypoplasia (d4) or left ventricle hypoplasia (d6). (E) Corresponding quantification of the right and left ventricle outer volumes in control (black dots) and mutant (pink squares) hearts. (F) Log2 ratio between the right and left ventricle outer volume. Means and standard deviations are shown. (G-H) Segmentation of the arterial trunks from 3D HREM images of a control (G) and *Nodal* mutants (H). The white dotted line indicates the diameter of each vessel at the level of the valve. Compared to balanced size of the arterial trunks in controls, *Nodal* mutants can show pulmonary trunk (h1) or ascending aorta (h2) hypoplasia. (I) Corresponding quantification of the pulmonary trunk and ascending aorta diameters in control (black dots) and mutant (pink squares) hearts. (J) Log2 ratio between the diameters of the pulmonary trunk and ascending aorta. Means and standard deviations are shown. The black dotted lines in B, E, F, I and J correspond to the 95% distribution interval of the control distribution. Scale bars: 500µm (A-C); 300µm (G). Ao, aorta; Caud., caudal; Cran., cranial; L, left; LV, left ventricle; MIP, maximum intensity projection; PT, pulmonary trunk; R, right; RV, right ventricle. See also Table S8.

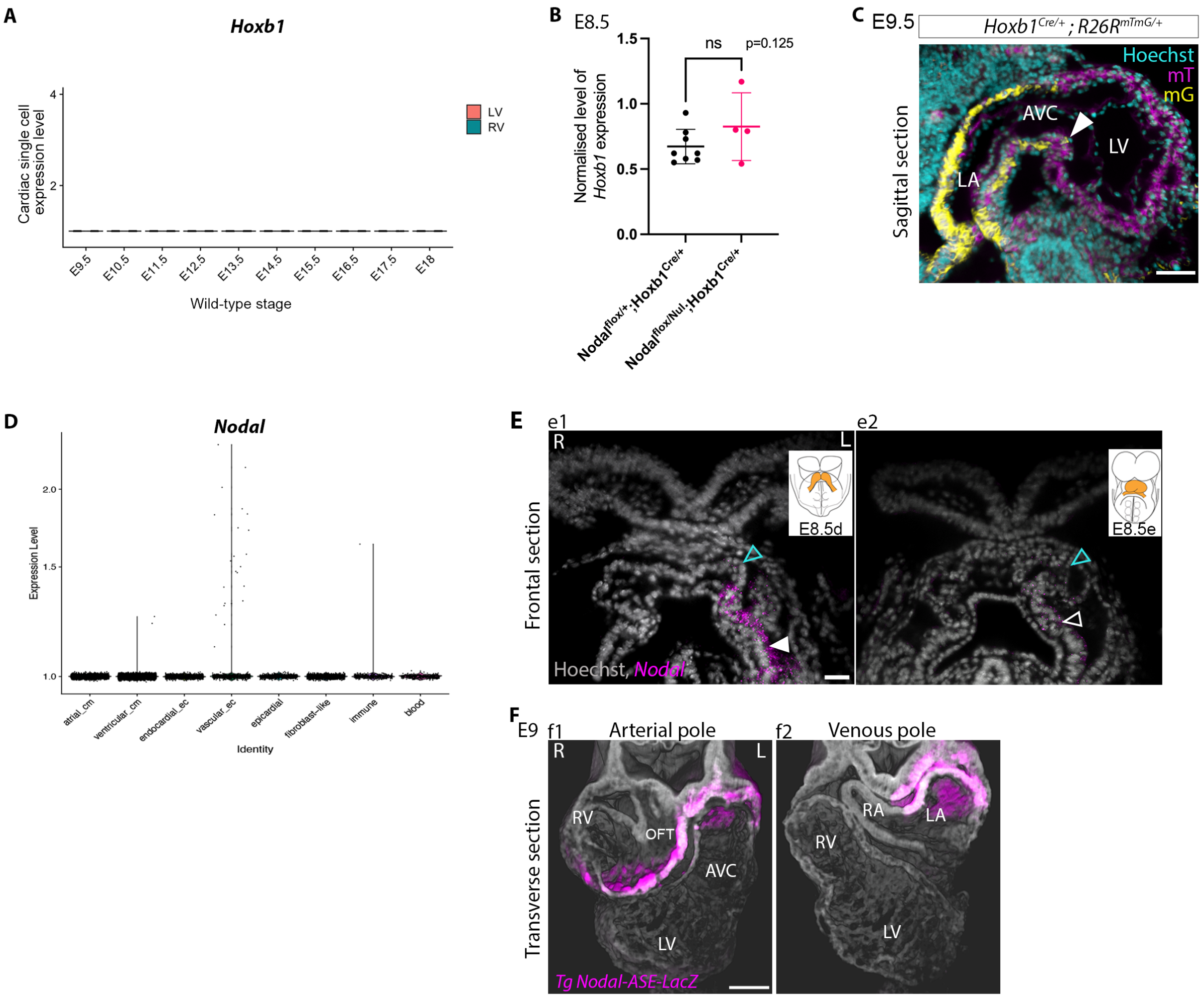

Fig. S4. Expression of *Hoxb1* and *Nodal* during mouse heart development

(A) Violin plot showing *Hoxb1* expression level in wild-type myocardial cells of the dataset Feng et al. (*54*) across stages (n=6,333 at E9.5, 557 at E10.5, 1,593 at E11.5, 1,249 at E12.5, 2,743 at E13.5, 970 at E14.5, 691 at E15.5, 378 at E16.5, 255 at E17.5, 2,125 at E18). (B) Normalised *Hoxb1* expression detected by RT-qPCR in *Nodal* homozygous mutants (red, n=4) compared to control heterozygotes (black, n=8) at E8.5g-h. (C) Genetic tracing of *Hoxb1* in *Hoxb1*$^{Cre/+}$;*R26R*$^{mTmG/+}$ embryos (n=3), showing rare mGFP-positive cells (white arrowhead), that have expressed *Hoxb1*, in the left ventricle myocardium at E9.5. A sagittal section of the 3D stack is shown. Most cells at E9.5 which have expressed *Hoxb1* are localised in the myocardium of the atrioventricular canal (AVC), and probably invade later the left ventricle as shown previously (*58*). (D) Violin plot showing the expression level of *Nodal* across cell types of the dataset Feng et al. (*54*), cumulatively between E9.5-E18 (n=3,292 atrial cm, 11,300 ventricular cm, 1,213 endocardial ec, 680 vascular ec, 779 epicardial, 1,837 fibroblast-like, 466 immune, 433 blood). ec, endothelial cells ; cm, cardiomyocytes. (E) Expression of *Nodal* in left heart precursors (filled white arrowhead) at E8.5d (e1) and repressed expression (empty arrowhead) at E8.5e (e2), detected by whole-mount RNAscope ISH and shown in frontal sections. Differentiated cardiomyocytes are indicated by blue arrowheads. (F) Genetic tracing with the *Tg Nodal-ASE-lacZ* line at E9, shown in HREM transverse sections. β-galactosidase-positive cells (pink), which have

expressed *Nodal*, do not contribute to the left ventricle, only marginally to the right ventricle outlet. L, left; LA, left atrium; LV, left ventricle; OFT, outflow tract; R, right; RA, right atrium; RV, right ventricle. Scale bars: 40µm (E), 100µm (C, F). See also Table S8.

Table S1. Spectrum of defects in *Nodal* conditional mutants, a model of heterotaxy with right isomerism.

DORV, double outlet right ventricle; TGA, transposition of the great arteries.

Table S2. Description of the mouse longitudinal cohort.

Table S3. Individual tracking in the uterine horns

The position in the uterine horn of embryos at E9.5 is labelled from R1 to Rn (right horn) and L1 to Ln (left horn). See also diagram in Fig. 1A. The same number of positions was found at E18.5. Although some individuals were lost during pregnancy (empty deciduum, degenerating fetus), this did not specifically affect mutants, which were collected at the expected Mendelian ratio (see Fig. S1J). The events of embryo death were detectable at E18.5, causing no loss of position in the uterine horn. In addition, anomalies seen at E9.5 (ex leftward looping), always matched a sample at E18.5 genotyped as mutant. In addition to our analysis of embryos immediately after ultrasound imaging (see Fig S2) validating positions in the uterine horns, the table overall supports accurate longitudinal pairing of individuals at E9.5 and E18.5.

Table S4. Individual phenotypes in the mouse longitudinal cohort.

Reporting of all parameters of the longitudinal cohort. Batches of collected fetuses are shown in light grey (litter and cardioplegia). Qualitative and quantitative parameters acquired in each individual of the cohort are shown at E9.5 (green) and E18.5. The most variable parameters at E18.5 are highlighted in orange (used for Multiple Correspondance Analysis in Fig. 1D and 6H), the low variable in pink (used together with orange parameters for Supervised classification models in Fig. 1E and 6I) and the nearly fully penetrant in blue. The 3 letter segmental nomenclature of congenital heart defects according to Van Praagh (*23*) is provided in white, reflecting the insertion of inferior caval veins, ventricle laterality and the malposition of the great arteries. A new classification arising from the study is highlighted in yellow. 6 samples, which had incomplete diagnosis of one parameter are highlighted in dark grey. Two Class 4 samples, with a medial left ventricle, potentially resembling the error in looping class diagnosis in the ground truth cohort are indicated in red. Ao, aorta; A/P, antero-posterior; AVC, atrioventricular canal ; DORV, double outlet right ventricle; L, left; LV, left ventricle ; N/A, the parameter could not be diagnosed ; PT, pulmonary trunk ; R, right; RV, right ventricle ; S/I supero-inferior; TGA, transposition of the great arteries ; VSD, ventricular septal defect.

Table S5. Individual phenotypes in heterotaxy patients.

Phenotypes were evaluated by CT scan and echocardiography. The most variable parameters are highlighted in orange, the low variable in pink and the nearly fully penetrant in blue. The 3 letter segmental nomenclature of congenital heart defects according to Van Praagh (*23*) is provided in white, reflecting the insertion of inferior caval veins, ventricle laterality and the malposition of the great arteries. Death is highlighted in yellow. All parameters are qualitative, except ventricle position, which was quantified as shown in Fig. 7. Ao, aorta; A/P, antero-posterior; AVC, atrioventricular canal ; DORV, double outlet right ventricle; L/R, left/right; LV, left ventricle ; PT, pulmonary trunk ; RV, right ventricle ; S/I supero-inferior; TGA, transposition of the great arteries ; VSD, ventricular septal defect.

Table S6. Death characteristics in the cohort of heterotaxy patients.

Asplenia or polysplenia were not enriched in the group with IVS malposition (36%, versus 38% in the group with normal IVS position), so that sepsis is more likely to be a complication of long term ICU. Fontan surgery does not imply any intracardiac surgical manipulation and ventricle malposition does not complexify this surgery. IVS, interventricular septum.

Table S7. List of oligonucleotides used for genotyping and RT-qPCR.

Table S8. Data.

Numerical data used to generate figure graphs along with statistical tests. Ao, ascending aorta; AVC, atrioventricular canal; CC, cranio-caudal; dOFT, distal outflow tract; DV, dorso-ventral; IVS, interventricular septum; LA, left atrium; LV, left ventricle; OFT, outflow tract; PT, pulmonary trunk; RA, right atrium; RV, right ventricle.

Movie S1. Examples of micro-ultrasound imaging in utero at E9.5.

The first sequence is a control embryo and the following show examples of *Nodal* mutant embryos of each looping class. Fast scans of embryos in utero from ventral to dorsal are provided. x/y resolution: 50 µm; speed of acquisition: 100 frames per second. In this series, image interpretation was controlled by dissection right after ultrasound imaging. Brightfield images of the same embryos after dissection are provided. LA, left atrium; LV, left ventricle; RA, right atrium; RV, right ventricle. See Figure S2.

Movie S2. Examples of micro-CT scans of visceral organs in situ at E18.5.

The first and second sequences show a control and *Nodal* mutant fetus, respectively. 3D rendering of the scan showing the external contour of the thoracic and abdominal cavities is provided, followed by a transparent whole mount view and then serial coronal sections. The identifier of the mutant sample is provided in the bottom right. Some bright artefactual aggregates of the contrast agent are seen in the vascular or cardiac lumen. x/y/z resolution, 20µm. See Figure S1B-G.

Movie S3. Examples of cardiac anatomy at E18.5 imaged by HREM.

The first and second sequences show a control and *Nodal* mutant fetus, respectively. 3D rendering showing the external heart contour is provided, followed by serial frontal sections. The identifier of the mutant sample is provided in the bottom right. Some bright artefactual aggregates of the contrast agent are seen in the vascular or cardiac lumen (red asterisks). x/y resolution, 2.9µm; z resolution, 2.34µm. See Figure S1B-C, H-I.

Source code S1. Matlab code used to evaluate cardiac chamber position in micro-ultrasound images in Fig. S2B

```
%Enter data
echo_data=zeros(8,3);


%Breakpoint->
plot3(echo_data(1:2,1),echo_data(1:2,2),echo_data(1:2,3))
hold on;plot3(echo_data(3:4,1),echo_data(3:4,2),echo_data(3:4,3))
hold
on;scatter3(echo_data(5:6,1),echo_data(5:6,2),echo_data(5:6,3),'fill','k')
hold
on;scatter3(echo_data(7:7,1),echo_data(7:7,2),echo_data(7:7,3),'fill','g')
hold
on;scatter3(echo_data(8:8,1),echo_data(8:8,2),echo_data(8:8,3),'fill','m')

%Scrip dashed line
plot3(echo_data(1:2,1),echo_data(1:2,2),echo_data(1:2,3),'--')
hold on;plot3(echo_data(3:4,1),echo_data(3:4,2),echo_data(3:4,3),'--')
hold
on;scatter3(echo_data(5:6,1),echo_data(5:6,2),echo_data(5:6,3),'fill','k')
hold
on;scatter3(echo_data(7:7,1),echo_data(7:7,2),echo_data(7:7,3),'fill','g')
hold
on;scatter3(echo_data(8:8,1),echo_data(8:8,2),echo_data(8:8,3),'fill','m')
```

Source code S2. Matlab code used to measure ventricle position based on the orientation of the interventricular septum in Table S4 and Fig. S3A-B

```
%Enter data
AB=zeros(1,3);
AC=zeros(1,3);
NT=zeros(2,3);
DV=zeros(2,3);

%Breakpoint

%Calcul Vn_norm et Calcul NC_norm
v=cross(AB,AC);
v_norm=v/norm(v);
NC=NT(1,:)-NT(2,:);
NC_norm=NC/norm(NC);

%Calcul DV-perp-norm
DV_line_perpendicular_to_NT;
DV_perp=DV_final(1,:)-DV_final(2,:);
DV_perp_norm=DV_perp/norm(DV_perp);

%Calcul Vproj_norm
Vproj=v_norm-dot(v_norm,DV_perp_norm).*DV_perp_norm
Vproj_norm=Vproj/norm(Vproj);

%Calcul angle in radian
acos(dot(Vproj_norm,NC_norm))

%Breakpoint

% calcul vectors in other plans
LR=cross(NC,DV_perp)
LR_norm=LR/norm(LR)

%Calcul Vproj_lr : v proj on sagital plan
Vproj_lr=v_norm-dot(v_norm,LR_norm).*LR_norm
Vproj_lr_norm=Vproj_lr/norm(Vproj_lr)

%Calcul angle in radian on sagital plan
acos(dot(Vproj_lr_norm,NC_norm))

% Breakpoint

% calcul 3 axes components
dot(v_norm,DV_perp_norm)
dot(v_norm,LR_norm)
dot(v_norm,NC_norm)
```

Web-interface : Longitudinal analysis of heterotaxy in *Nodal* mouse mutants

https://htx.pasteur.cloud

This page contains interactive graphs and statistical analyses of the cohort.

REFERENCES AND NOTES

1. L. Houyel, S. M. Meilhac, Heart development and congenital structural heart defects. *Annu. Rev. Genomics Hum. Genet.* **22**, 257–284 (2021).

2. A. Guimier, G. C. Gabriel, F. Bajolle, M. Tsang, H. Liu, A. Noll, M. Schwartz, R. El Malti, L. D. Smith, N. T. Klena, G. Jimenez, N. A. Miller, M. Oufadem, A. Moreau de Bellaing, H. Yagi, C. J. Saunders, C. N. Baker, S. Di Filippo, K. A. Peterson, I. Thiffault, C. Bole-Feysot, L. D. Cooley, E. G. Farrow, C. Masson, P. Schoen, J.-F. Deleuze, P. Nitschké, S. Lyonnet, L. de Pontual, S. A. Murray, D. Bonnet, S. F. Kingsmore, J. Amiel, P. Bouvagnet, C. W. Lo, C. T. Gordon, MMP21 is mutated in human heterotaxy and is required for normal left-right asymmetry in vertebrates. *Nat. Genet.* **47**, 1260–1263 (2015).

3. A. E. Lin, S. Krikov, T. Riehle-Colarusso, J. L. Frías, J. Belmont, M. Anderka, T. Geva, K. D. Getz, L. D. Botto, Laterality defects in the national birth defects prevention study (1998–2007): Birth prevalence and descriptive epidemiology. *Am. J. Med. Genet. A* **164**, 2581–2591 (2014).

4. T. G. Saba, G. C. Geddes, S. M. Ware, D. N. Schidlow, P. J. del Nido, N. S. Rubalcava, S. K. Gadepalli, T. Stillwell, A. Griffiths, L. M. Bennett Murphy, A. T. Barber, M. W. Leigh, N. Sabin, A. J. Shapiro, A multi-disciplinary, comprehensive approach to management of children with heterotaxy. *Orphanet J. Rare Dis.* **17**, 351 (2022).

5. S. Van Praagh, Cardiac malpositions and the heterotaxy syndromes, in *Nadas' Pediatric Cardiology*, J. F. Keane, J. E. Lock, D. C. Fyler, Eds. (Saunders-Elsevier, Philadelphia, PA, 2006), pp. 675–695.

6. E. M. Hagen, R. J. Sicko, D. M. Kay, S. L. Rigler, A. Dimopoulos, S. Ahmad, M. H. Doleman, R. Fan, P. A. Romitti, M. L. Browne, M. Caggana, L. C. Brody, G. M. Shaw, L. L. Jelliffe-Pawlowski, J. L. Mills, Copy-number variant analysis of classic heterotaxy highlights the importance of body patterning pathways. *Hum. Genet.* **135**, 1355–1364 (2016).

7. C. Meno, Y. Saijoh, H. Fujii, M. Ikeda, T. Yokoyama, M. Yokoyama, Y. Toyoda, H. Hamada, Left–right asymmetric expression of the TGFβ-family member lefty in mouse embryos. *Nature* **381**, 151–155 (1996).

8. S. Nonaka, Y. Tanaka, Y. Okada, S. Takeda, A. Harada, Y. Kanai, M. Kido, N. Hirokawa, Randomization of left–right asymmetry due to loss of nodal cilia generating leftward flow of extraembryonic fluid in mice lacking KIF3B motor protein. *Cell* **95**, 829–837 (1998).

9. J. Brennan, D. P. Norris, E. J. Robertson, Nodal activity in the node governs left-right asymmetry. *Genes Dev.* **16**, 2339–2344 (2002).

10. J. McGrath, S. Somlo, S. Makova, X. Tian, M. Brueckner, Two populations of node monocilia initiate left-right asymmetry in the mouse. *Cell* **114**, 61–73 (2003).

11. T. A. Katoh, T. Omori, K. Mizuno, X. Sai, K. Minegishi, Y. Ikawa, H. Nishimura, T. Itabashi, E. Kajikawa, S. Hiver, A. H. Iwane, T. Ishikawa, Y. Okada, T. Nishizaka, H. Hamada, Immotile cilia mechanically sense the direction of fluid flow for left-right determination. *Science* **379**, 66–71 (2023).

12. N. A. Brown, L. Wolpert, The development of handedness in left/right asymmetry. *Development* **109**, 1–9 (1990).

13. J.-F. Le Garrec, J. N. Domínguez, A. Desgrange, K. D. Ivanovitch, E. Raphaël, J. A. Bangham, M. Torres, E. Coen, T. J. Mohun, S. M. Meilhac, A predictive model of asymmetric morphogenesis from 3D reconstructions of mouse heart looping dynamics. *eLife* **6**, e28951 (2017).

14. A. Desgrange, J.-F. Le Garrec, S. Bernheim, T. H. Bønnelykke, S. M. Meilhac, Transient nodal signaling in left precursors coordinates opposed asymmetries shaping the heart loop. *Dev. Cell* **55**, 413–431.e6 (2020).

15. W. M. Layton Jr., M. D. Manasek, D. M. D. Manasek, Cardiac looping in early iv/iv mouse embryos, in *Etiology and Morphogenesis of Congenital Heart Disease*, R. Van Praagh, Ed. (Futura Pub. Co, New York, 1980), pp. 109–126.

16. Y.-T. Yan, K. Gritsman, J. Ding, R. D. Burdine, J. D. Corrales, S. M. Price, W. S. Talbot, A. F. Schier, M. M. Shen, Conserved requirement for EGF-CFC genes in vertebrate left-right axis formation. *Genes Dev.* **13**, 2527–2537 (1999).

17. M.-F. Lu, C. Pressman, R. Dyer, R. L. Johnson, J. F. Martin, Function of Rieger syndrome gene in left–right asymmetry and craniofacial development. *Nature* **401**, 276–278 (1999).

18. A. M. Hanning, M. E. Quinn, S. M. Ware, Heterotaxy-spectrum heart defects in *Zic3* hypomorphic mice. *Pediatr. Res.* **74**, 494–502 (2013).

19. F. T. Lewis, M. E. Abbott, Reversed torsion of the ventricular bend of the embryonic heart in the explanation of certain forms of cardiac anomaly. *Bull. Int. Assoc. Med. Mus.* **6**, 111 (1916).

20. M. V. de la Cruz, G. Anselmi, F. Cisneros, M. Reinhold, B. Portillo, J. Espino-Vela, An embryologic explanation for the corrected transposition of the great vessels: Additional description of the main anatomic features of this malformation and its varieties. *Am. Heart J.* **57**, 104–117 (1959).

21. R. Van Praagh, P. A. Ongley, H. J. C. Swan, Anatomic types of single or common ventricle in man. *Am. J. Cardiol.* **13**, 367–386 (1964).

22. R. Van Praagh, S. Van Praagh, P. Vlad, J. D. Keith, Anatomic types of congenital dextrocardia. *Am. J. Cardiol.* **13**, 510–531 (1964).

23. R. Van Praagh, The segmental approach to diagnosis in congenital heart disease. *Birth Defects Orig. Artic. Ser.* **8**, 4–23 (1972).

24. A. Desgrange, J. Lokmer, C. Marchiol, L. Houyel, S. M. Meilhac, Standardised imaging pipeline for phenotyping mouse laterality defects and associated heart malformations, at multiple scales and multiple stages. *Dis. Model. Mech.* **12**, dmm038356 (2019).

25. R. Van Praagh, P. M. Weinberg, S. Van Praagh. Malpositions of the heart, in *Heart Disease in Infants, Children and Adolescents*, A. J. Moss, F. H. Adams, G. E. Emmanouilides, Eds. (Williams and Wilkins, Baltimore, ed. 2, 1977), pp. 395–399.

26. S. Forlani, K. A. Lawson, J. Deschamps, Acquisition of Hox codes during gastrulation and axial elongation in the mouse embryo. *Development* **130**, 3807–3819 (2003).

27. N. Bertrand, M. Roux, L. Ryckebüsch, K. Niederreither, P. Dollé, A. Moon, M. Capecchi, S. Zaffran, Hox genes define distinct progenitor sub-domains within the second heart field. *Dev. Biol.* **353**, 266–274 (2011).

28. K. Minegishi, B. Rothé, K. R. Komatsu, H. Ono, Y. Ikawa, H. Nishimura, T. A. Katoh, E. Kajikawa, X. Sai, E. Miyashita, K. Takaoka, K. Bando, H. Kiyonari, T. Yamamoto, H. Saito, D. B. Constam, H. Hamada, Fluid flow-induced left-right asymmetric decay of Dand5 mRNA in the mouse embryo requires a Bicc1-Ccr4 RNA degradation complex. *Nat. Commun.* **12**, 4071 (2021).

29. Y. Saijoh, S. Oki, S. Ohishi, H. Hamada, Left–right patterning of the mouse lateral plate requires nodal produced in the node. *Dev. Biol.* **256**, 160–172 (2003).

30. T. Nakamura, N. Mine, E. Nakaguchi, A. Mochizuki, M. Yamamoto, K. Yashiro, C. Meno, H. Hamada, Generation of robust left-right asymmetry in the mouse embryo requires a self-enhancement and lateral-inhibition system. *Dev. Cell* **11**, 495–504 (2006).

31. M. T. M. Mommersteeg, W. M. H. Hoogaars, O. W. J. Prall, C. de Gier-de Vries, C. Wiese, D. E. W. Clout, V. E. Papaioannou, N. A. Brown, R. P. Harvey, A. F. M. Moorman, V. M. Christoffels, Molecular pathway for the localized formation of the sinoatrial node. *Circ. Res.* **100**, 354–362 (2007).

32. G. Ammirabile, A. Tessari, V. Pignataro, D. Szumska, F. Sutera Sardo, J. Benes Jr., M. Balistreri, S. Bhattacharya, D. Sedmera, M. Campione, Pitx2 confers left morphological, molecular, and functional identity to the sinus venosus myocardium. *Cardiovasc. Res.* **93**, 291–301 (2012).

33. P. S. Zammit, R. G. Kelly, D. Franco, N. Brown, A. F. M. Moorman, M. E. Buckingham, Suppression of atrial myosin gene expression occurs independently in the left and right ventricles of the developing mouse heart. *Dev. Dyn.* **217**, 75–85 (2000).

34. D. Franco, R. Kelly, A. F. M. Moorman, W. H. Lamers, M. Buckingham, N. A. Brown, MLC3F transgene expression in iv mutant mice reveals the importance of left-right signalling

pathways for the acquisition of left and right atrial but not ventricular compartment identity. *Dev. Dyn.* **221**, 206–215 (2001).

35. S. Zaffran, R. G. Kelly, S. M. Meilhac, M. E. Buckingham, N. A. Brown, Right ventricular myocardium derives from the anterior heart field. *Circ. Res.* **95**, 261–268 (2004).

36. Y. Sun, X. Liang, N. Najafi, M. Cass, L. Lin, C.-L. Cai, J. Chen, S. M. Evans, Islet 1 is expressed in distinct cardiovascular lineages, including pacemaker and coronary vascular cells. *Dev. Biol.* **304**, 286–296 (2007).

37. S. Bernheim, A. Borgel, J.-F. Le Garrec, E. Perthame, A. Desgrange, C. Michel, L. Guillemot, S. Sart, C. N. Baroud, W. Krezel, F. Raimondi, D. Bonnet, S. Zaffran, L. Houyel, S. M. Meilhac, Identification of Greb1l as a genetic determinant of crisscross heart in mice showing torsion of the heart tube by shortage of progenitor cells. *Dev. Cell* **58**, 2217–2234.e8 (2023).

38. T. H. Bønnelykke, M.-A. Chabry, E. Perthame, G. Dombrowsky, F. Berger, S. Dittrich, M.-P. Hitz, A. Desgrange, S. M. Meilhac, Notch3 is an asymmetric gene and a modifier of heart looping defects in Nodal mouse mutants. *PLOS Biol.* **23**, e3002598 (2025).

39. M. E. Piedra, J. M. Icardo, M. Albajar, J. C. Rodriguez-Rey, M. A. Ros, Pitx2 participates in the late phase of the pathway controlling left-right asymmetry. *Cell* **94**, 319–324 (1998).

40. D. Galli, J. N. Domínguez, S. Zaffran, A. Munk, N. A. Brown, M. E. Buckingham, Atrial myocardium derives from the posterior region of the second heart field, which acquires left-right identity as Pitx2c is expressed. *Development* **135**, 1157–1167 (2008).

41. F. Bajolle, S. Zaffran, R. G. Kelly, J. Hadchouel, D. Bonnet, N. A. Brown, M. E. Buckingham, Rotation of the myocardial wall of the outflow tract is implicated in the normal positioning of the great arteries. *Circ. Res.* **98**, 421–428 (2006).

42. T. J. Mohun, R. H. Anderson, 3D anatomy of the developing heart: Understanding ventricular septation. *Cold Spring Harb. Perspect. Biol.* **12**, a037465 (2020).

43. B. D. Sanketi, N. Zuela-Sopilniak, E. Bundschuh, S. Gopal, S. Hu, J. Long, J. Lammerding, S. Hopyan, N. A. Kurpios, Pitx2 patterns an accelerator-brake mechanical feedback through latent TGFβ to rotate the gut. *Science* **377**, eabl3921 (2022).

44. E. Szenker-Ravi, T. Ott, M. Khatoo, A. Moreau de Bellaing, W. X. Goh, Y. L. Chong, A. Beckers, D. Kannesan, G. Louvel, P. Anujan, V. Ravi, C. Bonnard, S. Moutton, P. Schoen, M. Fradin, E. Colin, A. Megarbane, L. Daou, G. Chehab, S. Di Filippo, C. Rooryck, J.-F. Deleuze, A. Boland, N. Arribard, R. Eker, S. Tohari, A. Y.-J. Ng, M. Rio, C. T. Lim, B. Eisenhaber, F. Eisenhaber, B. Venkatesh, J. Amiel, H. R. Crollius, C. T. Gordon, A. Gossler, S. Roy, T. Attie-Bitach, M. Blum, P. Bouvagnet, B. Reversade, Discovery of a genetic module essential for assigning left–right asymmetry in humans and ancestral vertebrates. *Nat. Genet.* **54**, 62–72 (2021).

45. M. L. Jacobs, Complications associated with heterotaxy syndrome in Fontan patients. *Semin. Thorac. Cardiovasc. Surg. Pediatr. Card. Surg. Annu.* **5**, 25–35 (2002).

46. J. P. Jacobs, R. H. Anderson, P. M. Weinberg, H. L. Walters III, C. I. Tchervenkov, D. D. Duca, R. C. G. Franklin, V. D. Aiello, M. J. Béland, S. D. Colan, J. William Gaynor, O. N. Krogmann, H. Kurosawa, B. Maruszewski, G. Stellin, M. J. Elliott, The nomenclature, definition and classification of cardiac structures in the setting of heterotaxy. *Cardiol. Young* **17**, 1–28 (2007).

47. C. C. Lu, E. J. Robertson, Multiple roles for Nodal in the epiblast of the mouse embryo in the establishment of anterior-posterior patterning. *Dev. Biol.* **273**, 149–159 (2004).

48. M. El Beheiry, C. Godard, C. Caporal, V. Marcon, C. Ostertag, O. Sliti, S. Doutreligne, S. Fournier, B. Hajj, M. Dahan, J.-B. Masson, DIVA: Natural navigation inside 3D images using virtual reality. *J. Mol. Biol.* **432**, 4745–4749 (2020).

49. A. Crucean, D. E. Spicer, J. T. Tretter, T. J. Mohun, R. H. Anderson, Revisiting the anatomy of the right ventricle in the light of knowledge of its development. *J. Anat.* **244**, 297–311 (2024).

50. B. Habib Geryes, R. Calmon, D. Khraiche, N. Boddaert, D. Bonnet, F. Raimondi, Radiation dose reduction in paediatric coronary computed tomography: Assessment of effective dose and image quality. *Eur. Radiol.* **26**, 2030–2038 (2016).

51. S. Lê, J. Josse, F. Husson, FactoMineR: An R package for multivariate analysis. *J. Stat. Softw.* **25**, 1–18 (2008).

52. M. Greenacre, J. Blasius, *Multiple Correspondence Analysis and Related Methods* (CRC Press, 2006).

53. M. N. Wright, A. Ziegler, ranger: A fast implementation of random forests for high dimensional data in C++ and R. *J. Stat. Softw.* **77**, 1–17 (2017).

54. W. Feng, A. Bais, H. He, C. Rios, S. Jiang, J. Xu, C. Chang, D. Kostka, G. Li, Single-cell transcriptomic analysis identifies murine heart molecular features at embryonic and neonatal stages. *Nat. Commun.* **13**, 7960 (2022).

55. D. P. Norris, E. J. Robertson, Asymmetric and node-specific nodal expression patterns are controlled by two distinct cis-acting regulatory elements. *Genes Dev.* **13**, 1575–1588 (1999).

56. B. R. Arenkiel, G. O. Gaufo, M. R. Capecchi, Hoxb1 neural crest preferentially form glia of the PNS. *Dev. Dyn.* **227**, 379–386 (2003).

57. M. D. Muzumdar, L. Luo, H. Zong, Modeling sporadic loss of heterozygosity in mice by using mosaic analysis with double markers (MADM). *Proc. Natl. Acad. Sci. U.S.A.* **104**, 4495–4500 (2007).

58. W. T. J. Aanhaanen, J. F. Brons, J. N. Domínguez, M. S. Rana, J. Norden, R. Airik, V. Wakker, C. de Gier-de Vries, N. A. Brown, A. Kispert, A. F. M. Moorman, V. M. Christoffels, The *Tbx2*$^{+}$ primary myocardium of the atrioventricular canal forms the atrioventricular node and the base of the left ventricle. *Circ. Res.* **104**, 1267–1274 (2009).
